# The clinical effectiveness and safety of using epidermal growth factor, fibroblast growth factor and granulocyte-macrophage colony stimulating factor as therapeutics in acute skin wound healing: a systematic review and meta-analysis

**DOI:** 10.1093/burnst/tkac002

**Published:** 2022-03-07

**Authors:** Yating Wei, Jiangfeng Li, Yao Huang, Xun Lei, Lijun Zhang, Meifang Yin, Jiawen Deng, Xiaoyan Wang, Xiaobing Fu, Jun Wu

**Affiliations:** 1 Department of Burn and Plastic Surgery, Department of Wound Repair, Shenzhen Institute of Translational Medicine, the First Affiliated Hospital of Shenzhen University, Shenzhen Second People’s Hospital, Shenzhen, China; 2 Shenzhen University, Shenzhen, China; 3 School of Public Health and Management, Chongqing Medical University, Chongqing, China; 4 The First Affiliated Hospital, Sun Yat-sen University, Guangzhou, China; 5 Research Center for Wound Repair and Tissue Regeneration, Medical Innovation Research Department, The Key Laboratory of PLA Wound Repair and Tissue Regeneration, the Fourth Medical Center of PLA General Hospital, the PLA General Hospital, Beijing 100048, China

**Keywords:** Growth factors, Skin wounds, Meta-analysis, Wound healing

## Abstract

**Background:**

Promoting wound healing is crucial to restore the vital barrier function of injured skin. Growth factor products including epidermal growth factor (EGF), fibroblast growth factor (FGF) and granulocyte-macrophage colony stimulating factor (GM-CSF) have been used for decades although no systematic evaluation exists regarding their effectiveness and safety issues in treating acute skin wounds. This has resulted in a lack of guidelines and standards for proper application regimes. Therefore, this systematic review and meta-analysis was performed to critically evaluate the effectiveness and safety of these growth factors on skin acute wounds and provide guidelines for application regimes.

**Methods:**

We searched PubMed/Medline (1980–2020), Cochrane Library (1980–2020), Cochrane CENTRAL (from establishment to 2020), ClinicalTrials.gov (from establishment to 2020), Chinese Journal Full-text Database (CNKI, 1994–2020), China Biology Medicine disc (CBM, 1978–2019), Chinese Scientific Journal Database (VIP, 1989–2020) and Wanfang Database (WFDATA, 1980–2019). Randomized controlled trials (RCTs), quasi-RCTs and controlled clinical trials treating patients with acute skin wounds from various causes and with those available growth factors were included.

**Results:**

A total of 7573 papers were identified through database searching; 229 papers including 281 studies were kept after final screening. Administering growth factors significantly shortened the healing time of acute skin wounds, including superficial burn injuries [mean difference (MD) = −3.02; 95% confidence interval (CI):−3.31 ~ −2.74; *p <* 0.00001], deep burn injuries (MD = −5.63; 95% CI:−7.10 ~ −4.17; *p* < 0.00001), traumata and surgical wounds (MD = −4.50; 95% CI:−5.55 ~ −3.44; *p* < 0.00001). Growth factors increased the healing rate of acute skin wounds and decreased scar scores. The incidence of adverse reactions was lower in the growth factor treatment group than in the non-growth factor group.

**Conclusions:**

The studied growth factors not only are effective and safe for managing acute skin wounds, but also accelerate their healing with no severe adverse reactions.

Highlights

- This study is the first to comprehensively evaluate the effectiveness and safety of using growth factors as therapeutics in acute skin wounds healing.
- Compared with non-growth factor treatment, administering growth factors significantly shortened the healing time while increasing the healing rate of acute skin wounds with lower scar scores and fewer adverse reactions.

## Background

Skin maintains internal homeostasis and provides a barrier between our body and the outside environment [1]. Acute skin wounds break the barrier and expose the body to the risk of pathogen infections and fluid losses. Therefore, restoring skin integrity as soon as possible after wounding is the body’s most effective way to restore the environment’s balance, fight infections and prevent fluid and electrolyte disturbances from occurring. The speed of wound healing is of essential importance and can impact on the patient’s prognosis [2].

Several factors can influence the speed of wound healing, such as the growth factors secreted by activated local cells. Numerous studies have recognized and elaborated upon growth factors’ crucial roles in advancing angiogenesis, re-epithelialization, granulation tissue formation and inflammatory response regulation [3]. Until now, the growth factors reported to promote wound healing mainly include vascular endothelial growth factors (VEGFs), fibroblast growth factors (FGFs), platelet-derived growth factors (PDGFs), transforming growth factor-β1(TGF-β1), epidermal growth factors (EGFs), granulocyte-macrophage colony stimulating factor (GM-CSF), hepatocyte growth factor (HGF), etc. [3–6].

In 1971, Frati and Scarpa reported the treatment of mouse burns with EGF [7]. The first human recombinant FGF-2 was reported in 1988 [8]. In 1989, Brown *et al*. reported in the *New England Journal of Medicine* that epidermal growth factor significantly accelerated the rate of healing of partial thickness skin wounds in a randomized clinical trial [9]. The development of growth factor products targeted at promoting wound healing has been thriving ever since and the clinical application of growth factors has become popular. In 1998, Fu *et al*. reported the result of a randomized placebo-controlled trial investigating the effect of recombinant bovine basic fibroblast growth factor (rbFGF) on burns healing. The study showed that rbFGF effectively decreased the time and improved the quality of healing. These favorable results started a wider trend of using growth factors in wound management [10]. In 2007, Ma *et al*. reported the use of recombinant human acidic FGF (rh-aFGF) for treating deep partial-thickness burns and skin graft donor site through a randomized, multicenter, double-blind and placebo-controlled trial. The study demonstrated that rh-aFGF can promote the healing of both burn wounds and skin graft donor sites [11], which further strengthened the evidence of applying growth factor products to promote acute wound healing, including both burns and surgical wounds.

Currently, EGF, bFGF, aFGF and GM-CSF are approved growth factor products for use on acute skin wounds. During the past decades, the therapeutic use of these growth factors in acute wounds management has gradually become a customary practice in China, however, controversies have raged about the benefits and safety of the clinical implementation of distinct kinds of growth factor products. It is known that acute wounds naturally hold plenty of growth factors, which can stimulate cell proliferation and matrix production at the wound bed. Whether the growth factor receptors are saturated prior to the application of more growth factors to acute wounds is unknown. Secondly, deep acute wounds usually heal with hypertrophic scars. It is still unclear whether deep acute wounds heal with more (or less) severe scars under the use of growth factors. Moreover, in light of the economic costs and possible side-effects (such as carcinogenesis) of high local/systemic growth factor levels, it is unclear whether the practice of using exogenous growth factors for the therapy of acute wounds is a real necessity. In addition, whether growth factor treatments provide true benefits remains uncertain given their instability and short *in vivo* half-life [4,12,13].

Notably, a systematic evaluation of the effectiveness and safety of the available growth factor products used for acute skin wound therapy is missing. There is still the need to investigate whether the routine administration strategies used in clinical treatments suffice to guarantee the growth factor products’ benefits. To address these issues, we performed the present systematic review and meta-analysis to assess the clinical effectiveness and safety of all currently clinically available growth factor products in treating acute skin wounds as compared to non-growth factor treatments. The results of this study will supply the evidence to strengthen the future therapeutic use of growth factors in clinical settings.

**Table 1:** Inclusion and exclusion criteria

**Criteria**	**Inclusion**	**Exclusion**
Type of study	Randomized controlled trials (RCTs), quasi-RCTs, controlled clinical trials	Review; case study; mechanism study; research; development; preparation and storage of materials; animal experiment; marketing strategy; editorials; news; and newly registered clinical trials without any reported results
Participants	Patients with acute skin wounds from various causes (e.g. burns, trauma, surgery, etc.)	Patients with deep burns (third- and fourth-degree burns), bone wounds, mucosal wounds
Interventions	Treatment with growth factors (epidermal growth factor, basic fibroblast growth, acidic fibroblast growth factor. granulocyte-macrophage colony stimulating factor)	Growth factor not used for wound treatment
Controls	Any other non-growth factor treatment; placebo; blank control	Comparison before and after their administration of the clinical results among different growth factors
Outcomes	Effectiveness indicators including wound healing time; wound healing rate; infection rate; pain score; pain intensity level; etc. Safety indicators referring to the adverse reactions rate, including skin allergy and pruritus	Long-term follow-up results such as related to quality of life. The growth factor levels set as treatment outcomes

## Methods

This systematic review was conducted according to the guidelines for Preferred Reporting Items for Systematic Reviews and Meta-analyses (PRISMA) [14]. It was based on the planned Participants, Intervention, Control, Outcome and Study design (PICOS) elements.

### Search strategy

The searched databases included: PubMed/Medline (1980–2020); Cochrane Library (1980–2020); Cochrane CENTRAL (from establishment to 2020); ClinicalTrials.gov (from establishment to 2020); Chinese Journal Full-text Database (CNKI, 1994–2020); China Biology Medicine disc (CBM, 1978–2019); Chinese Scientific Journal Database (VIP, 1989–2020); and Wanfang Database (WFDATA, 1980–2019). With the combination of subject words and free words, the search terms included two categories: (1) ‘epidermal growth factor’, ‘basic fibroblast growth factor’, ‘acid fibroblast growth factor’, and ‘granulocyte-macrophage colony stimulating factor’; and (2) ‘trauma’, ‘wound’, ‘burn’, and ‘surgery’. The logical relationship was created with ‘OR’ and ‘AND’; and the search formula was thereafter developed according to the characteristics of the different databases. For example, the search strategy for PubMed was: ((epidermal growth factor OR EGF) OR (basic fibroblast growth factor OR bFGF) OR (acid fibroblast growth factor OR aFGF) OR (granulocyte-macrophage colony stimulating factor OR GM-CSF)) AND ((superficial OR surgical OR burn) AND wounds)). A pre-retrieval process improved the searches strategy. In addition, we conducted a manual search of unpublished studies and conference materials, tracking also the references of the included literature. For the analysis we included studies reported in both Chinese and English.

### Inclusion and exclusion criteria

The inclusion and exclusion criteria are listed in Table 1.

#### Study selection

Two researchers independently read the titles and abstracts to exclude the literature that did not meet the inclusion criteria. As a further safeguard, the full texts of the literature that might have met the inclusion criteria were read and evaluated. At the same time, the following information was extracted: author, publication date, research type, characteristics of research objects, sample number, loss of or withdrawal from interview, intervention measures and measurement indicators, and more. For multiple studies published in the same literature, the required data were acquired according to their research contents. In the case of repetitive reports, the study included only the latest or the most comprehensive ones.

#### Quality evaluation

The quality of the included research method was evaluated via Jadad’s scale, which is an internationally recognized clinical trial scoring standard, as it includes data about random method, allocation concealment, blind use, loss of follow-up, withdrawal and outcome. The score range was 1–5 points, including 1–2 points for lower quality and 3–5 points for higher quality.

**Figure 1.**
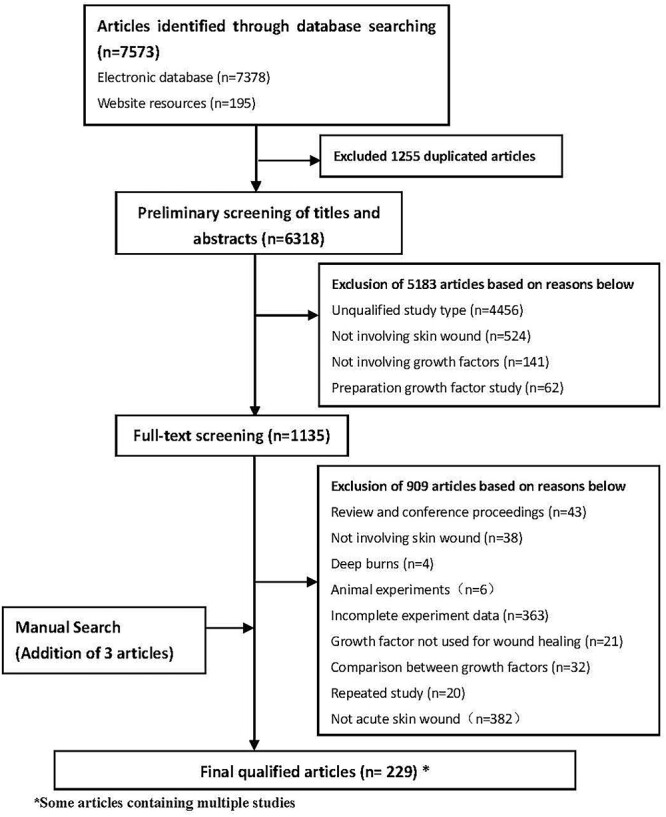
PRISMA flow diagram for inclusion or exclusion of studies used for this systematic review. *PRISMA* Preferred Reporting Items for Systematic Reviewsand Meta-analyses

#### Meta-analysis

The RevMan5.4 software recommended by Cochrane Collaboration served for meta-analysis. Subgroups considered types of wounds and outcome variables. The relative risk (RR) consisted of the joint effect size for the counting data, while the weighted mean difference (WMD) was used for the measurement data. All effects were conveyed with their 95% confidence interval (CI). Results heterogeneity was assessed by the chi square test. When the homogeneity of each study was statistically significant (*p* > 0.1, *I*^2^ < 50%), the fixed effect model was used; otherwise, the random effect model was used. Subgroup results from single studies were noted down.

## Results

### Study selection and characteristics

In total, our preliminary screening selected 7573 papers. After screening titles, abstracts and full-texts (Figure 1) we kept 229 papers including 281 studies, which consisted of 207 randomized controlled trials (RCTs) and 74 clinical controlled trials (CCTs) with a total of 30 562 patients. The basic characteristics of the included studies and the results of the methodological quality evaluations are shown in Table 2 [10,11,15–241]. All the growth factors in these studies were applied topically. In all studies, the patients’ basic characteristics were comparable (*p* > 0.05) between intervention groups and control groups.

**Figure 2.**
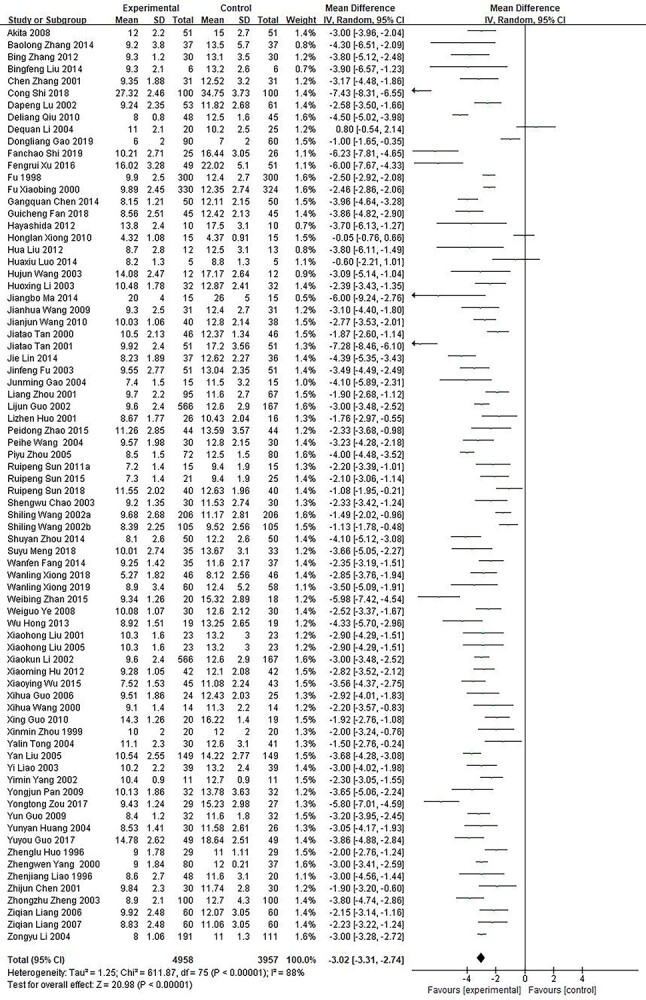
Comparative meta-analysis of the healing time of superficial second-degree burn wounds. *CI* confidence interval, *MD* mean difference

**Table 2:** Characteristics of included studies

**Author**	**Year**	**Study Design**	**Country**	**Wound Type**	**Sample size** **(Treatment)**	**Sample size** **(Control)**	**Jadad’s** **Score**
Pan *et al.* [15]	2009	CCT	China	Superficial Second-degree Burns	rhEGF+RI + 1%SD-Ag Cream(n = 64)	1%SD-Ag Cream(n = 64)	3
Wu *et al.* [16]	2013	CCT	China	Superficial Second-degree Burns	rhFGF+Zn-SD Gel(n = 19)	Zn-SD Gel(n = 19)	1
Guo *et al.* [17]	2017	RCT	China	Superficial Second-degree Burns	Er Huang Ointment +rhGM-CSF Gel(n = 49)	Ag-SD Cream(n = 49)	2
Ma *et al.* [18]	2014	CCT	China	Superficial Second-degree Burns	VSD + rb-bFGF(n = 9)	VSD(n = 9)	1
Huang *et al.* [19]	2004	RCT	China	Superficial Second-degree Burns	1% SD-Ag Cream+rhEGF(n = 30)	1% SD-Ag Cream(n = 26)	2
Li *et al.* [20]	2002	RCT	China	Superficial Second-degree Burns	rbFGF(n = 566)	0.9% NS(n = 167)	2
Chen *et al.* [21]	2001	RCT	China	Superficial Second-degree Burns	bFGF(n = 30)	SD-Ag Cream(n = 30)	2
Gao *et al.* [22]	2004	CCT	China	Superficial Second-degree Burns	bFGF(n = 15)	Blank(n = 15)	1
Huo *et al.* [23]	1996	CCT	China	Superficial Second-degree Burns	bFGF Spray(n = 29)	Blank(n = 29)	1
Li *et al.* [24]	2004	CCT	China	Superficial Second-degree Burns	bFGF(n = 191)	Blank(n = 191)	1
Hu *et al.* [25]	2012	RCT	China	Superficial Second-degree Burns	GM-CSF + AD-Ag Cream(n = 42)	SD-Ag Cream(n = 42)	2
Gong [26]	2007	RCT	China	Superficial Second-degree Burns	rhEGF Spray(n = 30)	Standard care(n = 30)	2
Luo [27]	2014	RCT	China	Superficial Second-degree Burns	1% Povidone iodine +rb-bFGF(n = 5)	1% Povidone iodine(n = 5)	2
Liao *et al.* [28]	1996	CCT	China	Superficial Second-degree Burns	EGF +1% SD-Ag(n = 48)	1% SD-Ag(n = 48)	2
Guo *et al.* [29]	2009	RCT	China	Superficial Second-degree Burns	rhEGF Hydrogel +Vaseline gauze(n = 32)	Vaseline gauze(n = 32)	2
Liu *et al.* [30]	2001	RCT	China	Superficial Second-degree Burns	rh-bFGF +1% SD-Ag(n = 23)	1% SD-Ag(n = 23)	1
Liu *et al.* [31]	2012	CCT	China	Superficial Second-degree Burns	rh-bFGF+1% SD-Ag(n = 12)	1% SD-Ag(n = 13)	1
Gao *et al.* [32]	2019	CCT	China	Superficial Second-degree Burns	rh-EGF Spray+Burn Cream(n = 90)	Povidone iodine(n = 60)	1
Li [33]	2003	RCT	China	Superficial Second-degree Burns	rhEGF+1% SD-Ag(n = 32)	1% SD-Ag(n = 32)	2
Liu *et al.* [34]	2005	CCT	China	Superficial Second-degree Burns	bFGF(n = 149)	Blank(n = 149)	1
Lin *et al.* [35]	2014	RCT	China	Superficial Second-degree Burns	rb-bFGF Gel(n = 37)	Blank(n = 36)	3
Guo *et al.* [36]	2002	CCT	China	Superficial Second-degree Burns	rb-bFGF Lyophilized powder(n = 566)	Standard Care(n = 167)	1
Fan *et al.* [37]	2018	RCT	China	Superficial Second-degree Burns	rb-bFGF Gel + Vaseline gauze(n = 45)	Vaseline gauze(n = 45)	2
Meng *et al.* [38]	2018	RCT	China	Superficial Second-degree Burns	rb-bFGF(n = 63)	Standard care(n = 63)	3
Guo *et al.* [39]	2010	RCT	China	Superficial Second-degree Burns	SD-Ag Cream+ rhEGF(n = 20)	SD-Ag cream(n = 19)	2
Fang *et al.* [40]	2014	RCT	China	Superficial Second-degree Burns	rhEGF(n = 35)	Blank(n = 37)	2
Liang *et al.* [41]	2007	CCT	China	Superficial Second-degree Burns	rhEGF(n = 60)	Normal saline(n = 60)	3
Liang *et al.* [42]	2006	CCT	China	Superficial Second-degree Burns	rhEGF(n = 60)	Normal saline(n = 60)	3
Huo *et al.* [43]	2001	CCT	China	Superficial Second-degree Burns	rhEGF+Topical antibiotics(n = 26)	Topical antibiotics(n = 26)	1
Fu *et al.* [44]	2003	CCT	China	Superficial Second-degree Burns	rhEGF(n = 51)	Blank(n = 51)	1
Liao *et al.* [45]	2003	RCT	China	Superficial Second-degree Burns	rhEGF+SD-Ag(n = 39)	1%SD-Ag cream(n = 39)	2
Li *et al.* [46]	2004	RCT	China	Superficial Second-degree Burns	rhEGF+Wuhuang oil(n = 20)	Wuhuang oil(n = 25)	2
Liu *et al.* [47]	2005	RCT	China	Superficial Second-degree Burns	rh-bFGF Lyophilized powder +1% SD-Ag(n = 23)	1% SD-Ag(n = 23)	2
Chao *et al.* [48]	2003	RCT	China	Superficial Second-degree Burns	rh-bFGF+ Vaseline gauze(n = 30)	Vaseline gauze(n = 30)	2
Guo [49]	2006	RCT	China	Superficial Second-degree Burns	rh-bFGF+1% SD-A(n = 24)	1% SD-Ag(n = 25)	2
Liu [50]	2014	RCT	China	Superficial Second-degree Burns	rh-bFGF(n = 6)	Standard care(n = 6)	2
Chen [51]	2014	CCT	China	Superficial Second-degree Burns	rh-aFGF(n = 50)	Normal saline(n = 50)	1
Sun *et al.* [52]	2011	RCT	China	Superficial Second-degree Burns	rh-aFGF(n = 15)	Normal saline(n = 15)	1
Qiu *et al.* [53]	2010	RCT	China	Superficial Second-degree Burns	bFGF+Bashi Cream(n = 48)	Vaseline gauze(n = 45)	2
Sun *et al.* [54]	2018	RCT	China	Superficial Second-degree Burns	rh-bFGF+Chitosan(n = 40)	Chitosan(n = 40)	3
Tan *et al.* [55]	2000	CCT	China	Superficial Second-degree Burns	bFGF+Topical antibiotics(n = 46)	Topical antibiotics(n = 46)	1
Song *et al.* [56]	2003	CCT	China	Superficial Second-degree Burns	Topical antibiotics+bFGF(n = 16)	Topical antibiotics(n = 18)	1
Tong *et al.* [57]	2004	CCT	China	Superficial Second-degree Burns	rhEGF(n = 30)	0.5% Complex iodine(n = 41)	1
Shi [58]	2019	RCT	China	Superficial Second-degree Burns	Nano-Ag + rh-EGF(n = 25)	Nano-Ag(n = 26)	3
Sun *et al.* [59]	2015	RCT	China	Superficial Second-degree Burns	aFGF(n = 21)	SD-Ag(n = 25)	3
Tan *et al.* [60]	2001	RCT	China	Superficial Second-degree Burns	rhEGF+5%SD-Ag(n = 51)	5%SD-Ag(n = 51)	2
Wang *et al.* [61]	2004	RCT	China	Superficial Second-degree Burns	rhEGF(n = 30)	Normal saline(n = 30)	2
Yang *et al.* [62]	2000	CCT	China	Superficial Second-degree Burns	bFGF(n = 80)	Blank(n = 80)	1
Wang *et al.* [63]	2000	CCT	China	Superficial Second-degree Burns	bFGF(n = 14)	Blank(n = 14)	1
Ye *et al.* [64]	2008	RCT	China	Superficial Second-degree Burns	rh-EGF + SD-Ag(n = 30)	SD-Ag(n = 30)	2
Wang *et al.* [65]	2010	RCT	China	Superficial Second-degree Burns	rh-EGF + Nano-Ag(n = 40)	0.5% PVP-I(n = 38)	2
Xiong *et al.* [66]	2010	CCT	China	Superficial Second-degree Burns	rh-EGF + Amnion(n = 15)	Amnion(n = 15)	1
Wang *et al.* [67]	2009	CCT	China	Superficial Second-degree Burns	rh-bFGF +Vaseline gauze(n = 31)	Vaseline gauze(n = 31)	1
Xiong [68]	2019	RCT	China	Superficial Second-degree Burns	rb-bFGF +SD-Ag(n = 60)	SD-Ag(n = 60)	2
Wang *et al.* [69]	2002	RCT	China	Superficial Second-degree Burns	rh-EGF Spray + SD-Ag(n = 206)	SD-Ag(n = 206)	3
Xu *et al.* [70]	2016	RCT	China	Superficial Second-degree Burns	rb-bFGF Hydrogel(n = 49)	Standard care(n = 51)	2
Xiong [71]	2018	RCT	China	Superficial Second-degree Burns	rh-EGF Hydrogel(n = 46)	Zhenshi Burn cream(n = 46)	2
Yang *et al.* [72]	2002	RCT	China	Superficial Second-degree Burns	rh-bFGF+SD-Ag(n = 11)	SD-Ag(n = 11)	2
Wang *et al.* [73]	2003	CCT	China	Superficial Second-degree Burns	rh-bFGF(n = 12)	Normal saline(n = 12)	1
Zhou *et al.* [74]	1999	RCT	China	Superficial Second-degree Burns	bFGF+Vaseline gauze(n = 20)	Vaseline gauze(n = 20)	2
Zhou *et al.* [75]	2005	RCT	China	Superficial Second-degree Burns	bFGF(n = 72)	Vaseline gauze(n = 80)	2
Zhan [76]	2015	RCT	China	Superficial Second-degree Burns	Nano-Ag + rh-EGF(n = 20)	Nano-Ag(n = 18)	2
Zhang *et al.* [77]	2014	RCT	China	Superficial Second-degree Burns	rb-bFGF Hydrogel(n = 37)	Topical antibiotics(n = 37)	2
Zhang *et al.* [78]	2001	CCT	China	Superficial Second-degree Burns	rb-bFGF(n = 31)	Blank(n = 31)	1
Zhao *et al.* [79]	2015	RCT	China	Superficial Second-degree Burns	rhEGF+Nano-Ag(n = 44)	Nano-Ag(n = 44)	3
Zou *et al.* [80]	2017	RCT	China	Superficial Second-degree Burns	rhEGF+Nano-Ag(n = 29)	Chlorhexidine(n = 27)	3
Zhou *et al.* [81]	2001	RCT	China	Superficial Second-degree Burns	rhEGF+SD-Ag Cream(n = 95)	SD-Ag Cream(n = 67)	3
Zhang [82]	2012	RCT	China	Superficial Second-degree Burns	rhEGF+ SD-Ag Cream(n = 30)	SD-Ag Cream(n = 30)	2
Zhen *et al.* [83]	2003	RCT	China	Superficial Second-degree Burns	rhEGF+ SD-Ag Cream(n = 100)	SD-Ag Cream(n = 100)	2
Zhou *et al.* [84]	2014	CCT	China	Superficial Second-degree Burns	rh-aFGF+ Hydrogen peroxide solution(n = 50)	Hydrogen peroxide solution(n = 50)	1
Wu *et al.* [85]	2015	RCT	China	Superficial Second-degree Burns	bFGF+ Hydrocolloid dressing(n = 45)	Vaseline gauze(n = 43)	3
Lu [86]	2002	CCT	China	Superficial Second-degree Burns	bFGF+1% SD-Ag Cream(n = 53)	1% SD-Ag Cream(n = 61)	1
Pan *et al.* [15]	2009	CCT	China	Deep Second-degree Burns	rhEGF+Insulin+1% SD-Ag(n = 56)	1% SD-Ag(n = 56)	3
Hu [87]	2013	RCT	China	Deep Second-degree Burns	bFGF Hydrogel+ Far infrared therapy(n = 22)	PVP-I Vaseline gauze + SD-Ag(n = 21)	3
Huang *et al.* [88]	2012	RCT	China	Deep Second-degree Burns	Local oxygen therapy +bFGF(n = 53)	Local oxygen therapy(n = 53)	2
Liu *et al.* [89]	2011	RCT	China	Deep Second-degree Burns	rhGM-CSF Hydrogel(n = 29)	Vaseline gauze(n = 29)	3
Hong *et al.* [16]	2013	CCT	China	Deep Second-degree Burns	bFGF+SD-Zn(n = 15)	SD-Zn(n = 15)	1
He *et al.* [90]	2018	RCT	China	Deep Second-degree Burns	Compound polymyxin B + EGF(n = 60)	Compound polymyxin B(n = 60)	3
Cheng *et al.* [91]	2011	RCT	China	Deep Second-degree Burns	rhGM-CSF Hydrogel+ Fulin honey(n = 56)	Placebo+SD-Ag Cream(n = 56)	4
Huang *et al.* [19]	2004	RCT	China	Deep Second-degree Burns	1% SD-Ag + rhEGF(n = 21)	1% SD-Ag (n = 20)	2
Li *et al.*n [20]	2002	RCT	China	Deep Second-degree Burns	rbFGF(n = 354)	Normal saline(n = 142)	2
Chen *et al.* [21]	2001	RCT	China	Deep Second-degree Burns	bFGF (n = 30)	SD-Ag Cream (n = 30)	2
Gao *et al.* [22]	2004	CCT	China	Deep Second-degree Burns	bFGF(n = 9)	Blank(n = 9)	1
Huo *et al.* [23]	1996	CCT	China	Deep Second-degree Burns	bFGF+1% SD-Ag Cream(n = 89)	1%1% SD-Ag Cream(n = 89)	1
Li *et al.* [24]	2004	CCT	China	Deep Second-degree Burns	bFGF(n = 54)	Blank(n = 54)	1
Chen *et al.* [92]	2013	RCT	China	Deep Second-degree Burns	Collegen+rh-EGF Hydrogel(n = 44)	SD-Ag(n = 44)	2
Chen *et al.* [93]	2012	RCT	China	Deep Second-degree Burns	MEBO +bFGF(n = 66)	MEBO(n = 69)	2
Liao *et al.* [94]	2018	RCT	China	Deep Second-degree Burns	Nano-Ag + rb-bFGF(n = 48)	Nano-Ag(n = 48)	3
Li *et al.* [95]	2015	RCT	China	Deep Second-degree Burns	Nano-Ag + rhEGF Hydrogel(n = 48)	Nano-Ag(n = 48)	1
Liao *et al.* [28]	1996	CCT	China	Deep Second-degree Burns	EGF(n = 32)	Normal saline(n = 20)	2
Han [96]	2018	RCT	China	Deep Second-degree Burns	rh-bFGF(n = 35)	Antibacterial dressing(n = 35)	3
Lin *et al.* [97]	2017	RCT	China	Deep Second-degree Burns	rhGM-CSF Hydrogel(n = 50)	1%SD-Ag + Vaseline gauze(n = 50)	3
Zeng [98]	2012	RCT	China	Deep Second-degree Burns	rhGM-CSF Hydrogel(n = 50)	PVP-I(n = 50)	3
Li [99]	2014	RCT	China	Deep Second-degree Burns	Insulin+rh-aFGF(n = 29)	Insulin(n = 29)	2
Meng *et al.* [100]	2005	RCT	China	Deep Second-degree Burns	rh-EGF+ SD-Ag(n = 56)	SD-Ag(n = 42)	2
Liu *et al.* [30]	2001	RCT	China	Deep Second-degree Burns	rh-bFGF+1% SD-Ag(n = 39)	1% SD-Ag(n = 39)	1
Liu *et al.* [31]	2012	CCT	China	Deep Second-degree Burns	rh-bFGF(n = 32)	1% SD-Ag(n = 35)	1
Gao *et al.* [32]	2019	CCT	China	Deep Second-degree Burns	rh-EGF(n = 153)	PVD-I(n = 147)	1
Liu *et al.* [34]	2005	CCT	China	Deep Second-degree Burns	bFGF(n = 399)	Blank(n = 399)	1
Lin *et al.* [35]	2014	RCT	China	Deep Second-degree Burns	rb-bFGF(n = 23)	PVD-I(n = 24)	3
Guo *et al.* [36]	2002	CCT	China	Deep Second-degree Burns	rb-bFGF(n = 354)	Standard care(n = 142)	1
Meng *et al.* [38]	2018	RCT	China	Deep Second-degree Burns	rb-bFGF(n = 28)	PVD-I(n = 30)	3
Guo *et al.* [39]	2010	RCT	China	Deep Second-degree Burns	SD-Ag + rhEGF(n = 20)	SD-Ag(n = 21)	2
Fang *et al.* [40]	2014	RCT	China	Deep Second-degree Burns	rhEGF(n = 32)	Blank(n = 30)	2
Liang *et al.* [41]	2007	CCT	China	Deep Second-degree Burns	rh-EGF(n = 60)	Normal saline(n = 60)	3
Liang *et al.* [42]	2006	CCT	China	Deep Second-degree Burns	rhEGF(n = 60)	Normal saline(n = 60)	3
Huo *et al.* [43]	2001	CCT	China	Deep Second-degree Burns	rhEGF(n = 16)	Normal saline(n = 16)	1
Han *et al.* [101]	2017	RCT	China	Deep Second-degree Burns	rhEGF+SD-Zn Gel(n = 34)	SD-Zn Gel(n = 34)	3
Chen *et al.* [102]	2017	CCT	China	Deep Second-degree Burns	rhEGF+ Mupirocin ointment(n = 300)	MEBO(n = 300)	1
Li [103]	2016	RCT	China	Deep Second-degree Burns	rhEGF Hydrogel(n = 32)	SD-Ag(n = 32)	2
Hua [104]	2019	RCT	China	Deep Second-degree Burns	rhEGF(n = 50)	MEBO(n = 50)	3
Fu *et al.* [44]	2003	CCT	China	Deep Second-degree Burns	rhEGF(n = 28)	Blank(n = 28)	1
Liao *et al.* [45]	2003	RCT	China	Deep Second-degree Burns	rhEGF(n = 21)	1% SD-Ag Cream(n = 21)	2
Li *et al.* [46]	2004	RCT	China	Deep Second-degree Burns	rhEGF+Wuhuang oil(n = 20)	Wuhuang oil(n = 25)	2
Liu *et al.* [47]	2005	RCT	China	Deep Second-degree Burns	rh-bFGF(n = 39)	Normal saline(n = 39)	2
Jin *et al.* [105]	2014	CCT	China	Deep Second-degree Burns	rh-bFGF(n = 36)	SD-Ag(n = 37)	1
Chao *et al.* [48]	2003	RCT	China	Deep Second-degree Burns	rh-bFGF(n = 50)	Vaseline gauze(n = 50)	2
Guo *et al.* [49]	2006	RCT	China	Deep Second-degree Burns	rh-bFGF(n = 16)	Normal saline(n = 15)	2
Liu *et al.* [50]	2014	RCT	China	Deep Second-degree Burns	Rh-bFGF(n = 4)	Standard care(n = 3)	2
Cai *et al.* [106]	2017	RCT	China	Deep Second-degree Burns	rhGM-CSF Hydrogel(n = 35)	Blank hydrogel(n = 35)	2
Lin [107]	2013	RCT	China	Deep Second-degree Burns	rhGM-CSF Hydrogel(n = 50)	Standard care(n = 40)	2
Chen *et al.* [51]	2014	CCT	China	Deep Second-degree Burns	rh-aFGF(n = 50)	PVD-I(n = 50)	1
Cai *et al.* [108]	2016	RCT	China	Deep Second-degree Burns	rh-aFGF+Vaseline gauze(n = 30)	Vaseline gauze(n = 30)	3
Sun *et al.* [52]	2011	RCT	China	Deep Second-degree Burns	rh-aFGF(n = 15)	Blank(n = 15)	1
Qiu *et al.* [53]	2010	RCT	China	Deep Second-degree Burns	rh-bFGF+Bashi cream(n = 38)	Vaseline gauze(n = 37)	2
Sui *et al.* [109]	2010	RCT	China	Deep Second-degree Burns	rb-bFGF+ Vaseline gauze(n = 132)	Vaseline gauze(n = 132)	2
Tong *et al.* [57]	2004	CCT	China	Deep Second-degree Burns	rhEGF(n = 32)	0.5% Complex iodine(n = 35)	1
Shi *et al.* [58]	2019	RCT	China	Deep Second-degree Burns	Nano-Ag + rh-EGF(n = 15)	Nano-Ag(n = 14)	3
Tong *et al.* [110]	2017	RCT	China	Deep Second-degree Burns	bFGF+SD-Zn(n = 53)	SD-Zn(n = 53)	2
Song *et al.* [111]	2018	RCT	China	Deep Second-degree Burns	rb-FGF Hydrogel(n = 37)	SD-Zn(n = 37)	3
Sun *et al.* [112]	2011	CCT	China	Deep Second-degree Burns	rh-aFGF(n = 24)	Normal saline(n = 22)	1
Sun *et al.* [112]	2011	CCT	China	Deep Second-degree Burns	bFGF(n = 20)	Normal saline (n = 22)	1
Qu [113]	2017	RCT	China	Deep Second-degree Burns	rhGM-CSF Hydrogel+Vaseline gauze(n = 48)	Vaseline gauze(n = 48)	3
Wang [114]	2014	RCT	China	Deep Second-degree Burns	rhGM-CSF(n = 15)	Placebo hydrogel(n = 15)	4
Xu [115]	2019	CCT	China	Deep Second-degree Burns	rh–bFGF(n = 15)	SD-Ag(n = 15)	1
Wang *et al.* [116]	2018	CCT	China	Deep Second-degree Burns	rhGM-CSF Hydrogel(n = 36)	Blank(n = 36)	1
Xu [117]	2017	RCT	China	Deep Second-degree Burns	EGF(n = 50)	Normal saline(n = 50)	3
Yan *et al.* [118]	2012	RCT	China	Deep Second-degree Burns	Silver ion dressing +rh-EGF hydrogel(n = 32)	Baikerui dressing(n = 32)	4
Wang *et al.* [61]	2004	RCT	China	Deep Second-degree Burns	rhEGF(n = 30)	Normal saline(n = 30)	2
Yang *et al.* [62]	2000	CCT	China	Deep Second-degree Burns	rh-bFGF(n = 37)	Blank(n = 37)	1
Xiong *et al.* [66]	2010	CCT	China	Deep Second-degree Burns	rh-EGF+ Amnion(n = 15)	Amnion(n = 15)	1
Yang *et al.* [119]	2018	RCT	China	Deep Second-degree Burns	Mupirocin ointment +GM-CSF hydrogel(n = 64)	Mupirocin ointment(n = 64)	3
Wang [67]	2009	CCT	China	Deep Second-degree Burns	rh-bFGF +Vaseline gauze(n = 31)	Vaseline gauze(n = 31)	1
Yang [120]	2014	RCT	China	Deep Second-degree Burns	GM-CSF Hydrogel(n = 38)	Vaseline gauze(n = 38)	3
Xiong *et al.* [68]	2019	RCT	China	Deep Second-degree Burns	rb-bFGF(n = 39)	SD-Ag(n = 41)	2
Wang *et al.* [69]	2002	RCT	China	Deep Second-degree Burns	rh-EGF Derivative(n = 138)	SD-Ag(n = 138)	3
Wen *et al.* [121]	2016	RCT	China	Deep Second-degree Burns	GM-CSF Hydrogel+ Mupirocin ointment(n = 25)	Mupirocin ointment(n = 25)	3
Yang *et al.* [122]	2018	RCT	China	Deep Second-degree Burns	rh-aFGF(n = 49)	Standard care (n = 45)	2
Xie *et al.* [123]	2018	RCT	China	Deep Second-degree Burns	rh-aFGF(n = 43)	Standard care(n = 43)	2
Wang [124]	2015	RCT	China	Deep Second-degree Burns	rb-bFGF(n = 78)	Nano-Ag(n = 78)	3
Wang [125]	2015	RCT	China	Deep Second-degree Burns	rb-bFGF Hydrogel(n = 60)	Vaseline gauze(n = 60)	2
You *et al.* [126]	2010	RCT	China	Deep Second-degree Burns	rhEGF Hydrogel(n = 16)	Placebo(n = 16)	4
Yang [127]	2013	RCT	China	Deep Second-degree Burns	rhEGF(n = 30)	SD-Ag(n = 30)	4
Yang *et al.* [72]	2002	RCT	China	Deep Second-degree Burns	rh-bFGF(n = 8)	SD-Ag(n = 8)	2
Wang *et al.* [73]	2003	CCT-	China	Deep Second-degree Burns	rh-bFGF(n = 20)	Normal saline(n = 20)	1
Zhang *et al.* [128]	2014	RCT	China	Deep Second-degree Burns	rb-bFGF+Nano-Ag(n = 40)	SD-Ag(n = 40)	2
Zhou *et al.* [74]	1999	RCT	China	Deep Second-degree Burns	bFGF(n = 20)	Vaseline gauze(n = 20)	2
Zhou *et al.* [75]	2005	RCT	China	Deep Second-degree Burns	bFGF(n = 80)	Vaseline gauze(n = 62)	2
Zhou *et al.* [129]	2015	RCT	China	Deep Second-degree Burns	EGF(n = 30)	Normal saline(n = 30)	3
Zhang *et al.* [130]	2010	RCT	China	Deep Second-degree Burns	rhEGF + SD-Ag(n = 30)	SD-Ag (n = 30)	2
Zhang *et al.* [131]	2011	RCT	China	Deep Second-degree Burns	rhEGF + SD-Ag(n = 30)	SD-Ag(n = 30)	2
Zhan *et al.* [76]	2015	RCT	China	Deep Second-degree Burns	Nano-Ag + rh-EGF(n = 19)	Nano-Ag(n = 18)	2
Zhou *et al.* [132]	2016	RCT	China	Deep Second-degree Burns	Nano-Ag + rb-bFGF(n = 15)	Nano-Ag(n = 15)	2
Zhao *et al.* [133]	2001	RCT	China	Deep Second-degree Burns	rb-bFGF(n = 52)	Vaseline gauze(n = 52)	2
Zhang [134]	2019	RCT	China	Deep Second-degree Burns	GM-CSF Hydrogel(n = 80)	Vaseline gauze(n = 80)	2
Zhang *et al.* [78]	2001	CCT	China	Deep Second-degree Burns	rb-bFGF(n = 80)	Blank(n = 80)	1
Zou *et al.* [80]	2017	RCT	China	Deep Second-degree Burns	rh-EGF + Nano-Ag(n = 27)	Chlorhexidine(n = 28)	3
Zhou *et al.* [81]	2001	RCT	China	Deep Second-degree Burns	rhEGF(n = 109)	Placebo(n = 76)	3
Zhang *et al.* [82]	2012	RCT	China	Deep Second-degree Burns	rhEGF+SD-Ag Cream(n = 38)	SD-Ag Cream(n = 38)	3
Zhang *et al.* [135]	2010	RCT	China	Deep Second-degree Burns	rh-EGF(n = 21)	Ag-Zn Cream(n = 16)	2
Zhang *et al.* [136]	2016	RCT	China	Deep Second-degree Burns	rhGM-CSF(n = 20)	Rifampicin(n = 20)	3
Zhou *et al.* [84]	2014	CCT	China	Deep Second-degree Burns	rh-aFGF(n = 45)	Blank(n = 45)	1
Deng [137]	2017	CCT	China	Deep Second-degree Burns	rhGM-CSF + SD-Ag(n = 33)	SD-Ag(n = 33)	1
Chen *et al.* [138]	2009	RCT	China	Deep Second-degree Burns	Fulin honey+rh-EGF Hydrogel(n = 60)	Povidone iodine(n = 60)	3
Liu *et al.* [139]	2016	RCT	China	Deep Second-degree Burns	rhGM-CSF(n = 177)	PVD-I(n = 181)	2
Yan *et al.* [140]	2016	RCT	China	Deep Second-degree Burns	rh-EGF Hydrogel + Nano-Ag(n = 40)	Nano-Ag(n = 40)	3
Jiao *et al.* [141]	2014	CCT	China	Deep Second-degree Burns	rhGM-CSF + SD-Ag(n = 15)	SD-Ag(n = 15)	1
Xia *et al.* [142]	2015	CCT	China	Deep Second-degree Burns	rhGM-CSF(n = 30)	Standard care(n = 28)	1
Ma *et al.* [143]	2008	RCT	China	Deep Second-degree Burns	rh-aFGF(n = 32)	Placebo(n = 32)	3
Shi *et al.* [144]	2018	RCT	China	Deep Second-degree Burns	Dragon blood powder +rb-bFGF Hydrogel(n = 100)	Jingwanhong ointment + Kangfuxin liquid(n = 100)	2
Wu *et al.* [145]	2012	RCT	China	Deep Second-degree Burns	Gentamicin + Heparin +bFGF Hydrogel(n = 63)	Gentamicin(n = 58)	2
Wu *et al.* [145]	2012	RCT	China	Deep Second-degree Burns	Gentamicin +Red light therapy+Heparin+bFGF Hydrogel(n = 60)	Gentamicin(n = 58)	2
Zhou *et al.* [146]	2016	RCT	China	Deep Second-degree Burns	rhGM-CSF+ Nano-Ag(n = 30)	Nano-Ag(n = 30)	3
Ge *et al.* [147]	2001	CCT	China	Trauma and Surgical Wound	bFGF(n = 53)	Furacilin + Vaseline gauze(n = 66)	1
Niu *et al.* [148]	2016	CCT	China	Trauma and Surgical Wound	rh-aFGF(n = 90)	Vaseline gauze(n = 90)	1
Dong [149]	2016	RCT	China	Trauma and Surgical Wound	bFGF + Mupifloxacin(n = 42)	Vaseline gauze(n = 42)	2
Chen *et al.* [150]	2017	RCT	China	Trauma and Surgical Wound	rh-EGF(n = 143)	Infrared radiation(n = 143)	3
Hao *et al.* [151]	2015	RCT	China	Trauma and Surgical Wound	Compound schizonepeta fumigation lotion+rh-bFGF(n = 165)	Kangfuxin liquid(n = 144)	2
Liu *et al.* [152]	2004	CCT	China	Trauma and Surgical Wound	bFGF(n = 58)	Vaseline gauze(n = 48)	1
Li *et al.* [153]	2013	RCT	China	Trauma and Surgical Wound	rh-EGF(n = 30)	40% Magnesium sulfate glycerin(n = 30)	3
Guo *et al.* [154]	2003	RCT	China	Trauma and Surgical Wound	bFGF(n = 68)	Furacilin + Vaseline gauze(n = 41)	2
Huang *et al.* [155]	2010	RCT	China	Trauma and Surgical Wound	bFGF(n = 30)	Standard care(n = 30)	2
Chen *et al.* [156]	2010	RCT	China	Trauma and Surgical Wound	bFGF(n = 20)	Gentamicin(n = 20)	3
Li *et al.* [157]	2015	RCT	China	Trauma and Surgical Wound	rb-FGF + ACRSC(n = 27)	ACRSC(n = 27)	2
Ge *et al.* [158]	2002	RCT	China	Trauma and Surgical Wound	bFGF(n = 87)	Furacilin + Vaseline gauze(n = 53)	2
Li *et al.* [159]	2002	CCT	China	Trauma and Surgical Wound	bFGF(n = 89)	Standard care(n = 84)	1
Li [160]	2016	RCT	China	Trauma and Surgical Wound	rh-EGF(n = 120)	TCM lotions(n = 120)	2
Fu *et al.* [161]	2015	RCT	China	Trauma and Surgical Wound	rh-EGF(n = 36)	Vaseline gauze(n = 36)	3
Qi *et al.* [162]	2009	CCT	China	Trauma and Surgical Wound	rh-EGF(n = 183)	0.1% Rivanol (n = 204)	1
Li *et al.* [163]	2012	RCT	China	Trauma and Surgical Wound	rh-EGF(n = 84)	Standard care(n = 83)	3
Li *et al.* [164]	2016	RCT	China	Trauma and Surgical Wound	Cosmetic suture + rh-EGF(n = 55)	Ordinary suture(n = 55)	2
Fan *et al.* [165]	2011	RCT	China	Trauma and Surgical Wound	rh-EGF(n = 50)	TCM gauze(n = 50)	3
Deng [166]	2008	RCT	China	Trauma and Surgical Wound	rh-EGF(n = 35)	TCM gauze(n = 35)	3
Liu *et al.* [167]	2019	RCT	China	Trauma and Surgical Wound	GM-CSF Hydrogel(n = 55)	Artificial dermis(n = 55)	3
Li *et al.* [168]	2015	RCT	China	Trauma and Surgical Wound	rh-bFGF(n = 25)	Sanqi Shengji ointment(n = 25)	3
Meng *et al.* [169]	2019	CCT	China	Trauma and Surgical Wound	rh-aFGF(n = 30)	Vaseline gauze(n = 30)	1
Huang *et al.* [170]	2018	RCT	China	Trauma and Surgical Wound	rh-bFGF(n = 29)	Fu Zhi Qing(n = 30)	3
He [171]	2015	RCT	China	Trauma and Surgical Wound	rh-bFGF(n = 40)	Vaseline gauze(n = 40)	2
Long *et al.* [172]	2014	RCT	China	Trauma and Surgical Wound	rb-bFGF +Arnebia oil guaze(n = 50)	Arnebia oil gauze(n = 50)	2
Guo *et al.* [173]	2018	RCT	China	Trauma and Surgical Wound	rb-bFGF(n = 40)	Standard care(n = 40)	2
Li *et al.* [174]	2018	RCT	China	Trauma and Surgical Wound	rh-EGF Hydrogel(n = 30)	Standard care(n = 30)	2
Li *et al.* [174]	2018	RCT	China	Trauma and Surgical Wound	rh-EGF Solution(n = 30)	Standard care(n = 30)	2
Jiang *et al.* [175]	2018	RCT	China	Trauma and Surgical Wound	rh-EGF(n = 24)	Standard care(n = 24)	2
Liu *et al.* [176]	2018	RCT	China	Trauma and Surgical Wound	rh-EGF(n = 45)	Vaseline gauze(n = 45)	3
Liao *et al.* [177]	2008	RCT	China	Trauma and Surgical Wound	rh-EGF(n = 60)	Vaseline gauze(n = 60)	2
Lu *et al.* [178]	2017	RCT	China	Trauma and Surgical Wound	rh-EGF Hydrogel(n = 68)	Normal saline(n = 68)	2
Huang *et al.* [179]	2004	CCT	China	Trauma and Surgical Wound	rh-EGF(n = 30)	PVD-I gauze(n = 30)	1
Lin *et al.* [180]	2019	RCT	China	Trauma and Surgical Wound	rh-bFGF(n = 50)	Blank(n = 50)	2
Liu *et al.* [181]	2018	RCT	China	Trauma and Surgical Wound	rh-aFGF(n = 30)	Normal saline(n = 30)	3
Jiang [182]	2006	CCT	China	Trauma and Surgical Wound	bFGF(n = 91)	Normal saline(n = 85)	1
Sun *et al.* [183]	2017	RCT	China	Trauma and Surgical Wound	bFGF+ Mupirocin ointment(n = 44)	Mupirocin ointment(n = 32)	2
Sun *et al.* [184]	2011	RCT	China	Trauma and Surgical Wound	Rh-aFG F(n = 22)	Vaseline gauze(n = 18)	2
Sun *et al.* [185]	2014	RCT	China	Trauma and Surgical Wound	rh-aFGF(n = 22)	Vaseline gauze(n = 16)	2
Sun *et al.* [186]	2009	CCT	China	Trauma and Surgical Wound	bFGF(n = 50)	Shengji Yuhong ointment(n = 46)	1
Shi *et al.* [187]	2016	RCT	China	Trauma and Surgical Wound	Erythromycin ointment +rh-EGF Hydrogel(n = 65)	Erythromycin ointment(n = 65)	3
Shi *et al.* [188]	2012	RCT	China	Trauma and Surgical Wound	rh-EGF Hydrogel(n = 53)	Vaseline gauze(n = 53)	2
Teng *et al.* [189]	2015	RCT	China	Trauma and Surgical Wound	rh-EGF Hydrogel(n = 22)	Standard care(n = 22)	2
You [190]	2019	RCT	China	Trauma and Surgical Wound	bFGF(n = 30)	Chlorophyll derivative(n = 30)	3
Wang [191]	2018	RCT	China	Trauma and Surgical Wound	rb-bFGF+Hydrosorb(n = 16)	Hydrosorb(n = 16)	2
Wang *et al.* [192]	2014	RCT	China	Trauma and Surgical Wound	rh-aFGF(n = 52)	Gelatin sponge(n = 52)	5
Wang *et al.* [193]	2008	RCT	China	Trauma and Surgical Wound	bFGF(n = 46)	Gentamicin(n = 50)	2
Wen *et al.* [194]	2005	RCT	China	Trauma and Surgical Wound	rh-EGF(n = 86)	1%PVD-I(n = 73)	2
Wang [195]	2016	RCT	China	Trauma and Surgical Wound	rh-EGF+ 2%Iodine(n = 50)	Anisodamine + Gentamicin + Insulin + Normal saline(n = 50)	2
Xu [196]	2019	RCT	China	Trauma and Surgical Wound	Cosmetic suture + rh-EGF(n = 30)	Cosmetic suture(n = 30)	2
Yao *et al.* [197]	2014	RCT	China	Trauma and Surgical Wound	rh-aFGF(n = 81)	Normal saline(n = 86)	2
Wu *et al.* [198]	2016	RCT	China	Trauma and Surgical Wound	rh-bFGF(n = 37)	PVD-I(n = 39)	3
Wang *et al.* [199]	2018	RCT	China	Trauma and Surgical Wound	rb-bFGF Hydrogel(n = 30)	Jiyuhong ointment(n = 30)	2
Wu *et al.* [200]	2004	RCT	China	Trauma and Surgical Wound	rbFGF(n = 36)	Blank(n = 36)	2
Xu *et al.* [201]	2000	RCT	China	Trauma and Surgical Wound	rbFGF(n = 69)	Normal saline(n = 20)	2
Wei [202]	2017	RCT	China	Trauma and Surgical Wound	rh-EGF + bFGF(n = 80)	rh-EGF(n = 80)	3
Xie *et al.* [203]	2013	RCT	China	Trauma and Surgical Wound	rh-EGF Hydrogel(n = 55)	Vaseline gauze(n = 55)	3
Wu *et al.* [204]	2004	RCT	China	Trauma and Surgical Wound	rh-EGF(n = 31)	Mayinglong ointment(n = 35)	2
Wang *et al.* [205]	2014	CCT	China	Trauma and Surgical Wound	EGF(n = 30)	Normal saline(n = 30)	1
Wu *et al.* [206]	2013	RCT	China	Trauma and Surgical Wound	aFGF(n = 58)	Titanoreine(n = 58)	3
Zhi *et al.* [207]	2007	RCT	China	Trauma and Surgical Wound	EGF(n = 54)	Vaseline gauze(n = 53)	2
Zhu *et al.* [208]	2006	CCT	China	Trauma and Surgical Wound	rh-EGF(n = 24)	Blank(n = 26)	1
Zhang *et al.* [209]	2015	CCT	China	Trauma and Surgical Wound	rh-EGF(n = 148)	PVD-I(n = 148)	1
Zhong *et al.* [210]	2015	RCT	China	Trauma and Surgical Wound	rh-EGF(n = 78)	Normal saline(n = 72)	2
Zhai *et al.* [211]	2010	RCT	China	Trauma and Surgical Wound	rb-bFGF(n = 23)	Vaseline gauze(n = 22)	2
Zhang *et al.* [212]	2007	RCT	China	Trauma and Surgical Wound	bFGF(n = 50)	Blank(n = 10)	2
Zhang *et al.* [213]	2001	CCT	China	Trauma and Surgical Wound	bFGF(n = 120)	Mupirocin ointment(n = 80)	1
Zhou *et al.* [214]	2011	RCT	China	Trauma and Surgical Wound	rb-bFGF(n = 64)	Longzhu ointment(n = 64)	2
Mei *et al.* [215]	2019	RCT	China	Trauma and Surgical Wound	rh-EGF + Cosmetic suture(n = 47)	Standard Care(n = 46)	2
Zhang *et al.* [216]	2012	RCT	China	Trauma and Surgical Wound	bFGF + Compound Sihuang liquid(n = 80)	Standard Care(n = 80)	3
Zhu *et al.* [217]	2012	RCT	China	Trauma and Surgical Wound	rh-EGF(n = 24)	Vaseline gauze(n = 24)	2
Zhou *et al.* [218]	2015	RCT	China	Trauma and Surgical Wound	rh-EGF Hydrogel(n = 56)	Metronidazole Ethacridine Lactate(n = 56)	2
Zhao *et al.* [219]	2019	RCT	China	Trauma and Surgical Wound	rh-EGF(n = 54)	Metronidazole(n = 54)	3
Zhu [220]	2007	CCT	China	Trauma and Surgical Wound	bFGF(n = 30)	5%PVD-I(n = 26)	1
Zhang [221]	2019	RCT	China	Trauma and Surgical Wound	rh-aFGF(n = 60)	Gelatin sponge(n = 60)	3
Zhang [222]	2004	RCT	China	Trauma and Surgical Wound	rb-bFGF(n = 65)	Shengji Yuhong ointment(n = 51)	2
Yun *et al.* [223]	2007	RCT	China	Trauma and Surgical Wound	bFGF(n = 61)	Standard care(n = 63)	2
Huang [224]	2017	RCT	China	Trauma and Surgical Wound	rh-EGF Hydrogel(n = 40)	Metronidazole(n = 40)	3
Xu [225]	2017	RCT	China	Trauma and Surgical Wound	EGF(n = 24)	PVD-I(n = 24)	3
Zhang *et al.* [226]	2017	RCT	China	Trauma and Surgical Wound	bFGF(n = 30)	Kangfuxin(n = 30)	2
Luo [227]	2018	RCT	China	Trauma and Surgical Wound	rh-bFGF(n = 30)	PVD-I(n = 30)	2
Wang [228]	2016	RCT	China	Trauma and Surgical Wound	GM-CSF Hydrogel(n = 30)	Metronidazole(n = 30)	2
Sun *et al.* [229]	2010	RCT	China	Trauma and Surgical Wound	rh-EGF Spray (n = 38)	Gentamicin(n = 20)	3
Fu *et al.* [230]	2000	CCT	China	Second Degree Burns	rb-FGF(n = 330)	Placebo(n = 324)	2
Ichiro *et al.* [231]	2007	CCT	Japan	Trauma and Surgical Wound	bFGF	Standard care	2
Yan *et al.* [232]	2017	RCT	China	Deep Second-degree Burns	rhGM-CSF(n = 95)	Placebo(n = 95)	3
Lin *et al.* [233]	2015	RCT	China	Deep Second-degree Burns	rhGM-CSF(n = 21)	Mupirocin ointment(n = 21)	2
Akita *et al.* [234]	2008	RCT	Japan	Superficial Second-degree Burns	bFGF(n = 51)	Vaseline gauze(n = 51)	2
Nie *et al.* [235]	2010	RCT	China	Deep Second-degree Burns	bFGF+Oxygen therapy(n = 44)	Oxygen therapy(n = 41)	2
Hayashida *et al.* [236]	2012	RCT	Japan	Superficial Second-degree Burns	bFGF(n = 10)	Placebo(n = 10)	2
Fu *et al.* [10]	1998	RCT	China	Second Degree Burns	bFGF(n = 300)	Placebo(n = 300)	2
Ma *et al.* [11]	2007	RCT	China	Deep Second-degree Burns	aFGF(n = 39)	Placebo(n = 39)	3
Wang *et al.* [237]	2002	RCT	China	Second Degree Burns	EGF(n = 105)	Placebo(n = 105)	2
Wang *et al.* [238]	2003	RCT	China	Deep Second-degree Burns	EGF(n = 37)	Placebo(n = 37)	2
Wang *et al.* [239]	2008	RCT	China	Deep Second-degree Burns	GM-CSF(n = 214)	Placebo(n = 107)	2
Yan Hong *et al.* [240]	2012	RCT	China	Deep Second-degree Burns	rhGM-CSF(n = 32)	Placebo(n = 33)	3
Zhang *et al.* [241]	2009	RCT	China	Deep Second-degree Burns	GM-CSF(n = 60)	Placebo(n = 30)	2

*ACRSC* avene cicalfate restorative skin cream, *CCT* controlled clinical trial, *EGF* epidermal growth factor, *FFG* fibroblast growth factor, *GM-CSF* granulocyte-macrophage colony stimulating factor, *MEBO* moist exposed burn ointment, *PVP-I* polyvinyl pyrrolidone, *PVD-I* povidone iodine, *rbFGF* recombinant bovine basic fibroblast growth factor; *rh-aFGF* recombinant human acidic fibroblast growth factor, *RCT* randomized controlled trial, *TCM* traditional chinese medicine

### Healing time comparison of second-degree burn wounds

A total of 76 studies [10,15–25,27–55,57–86,144,230,234,236,237] enrolling 8915 cases compared the healing time of superficial second-degree burn wounds between growth factor and other non-growth factor treatments. The results showed the presence of statistical heterogeneity (*p* < 0.00001; *I*^2^ = 88%). Therefore, the random effect model was used for meta-analysis (Figure 2). The results showed that the wound healing time was 3.02 days shorter in the growth factor group than in the control group (MD = −3.02; 95% CI:−3.31 ~ −2.74; *p* < 0.00001).

**Figure 3.**
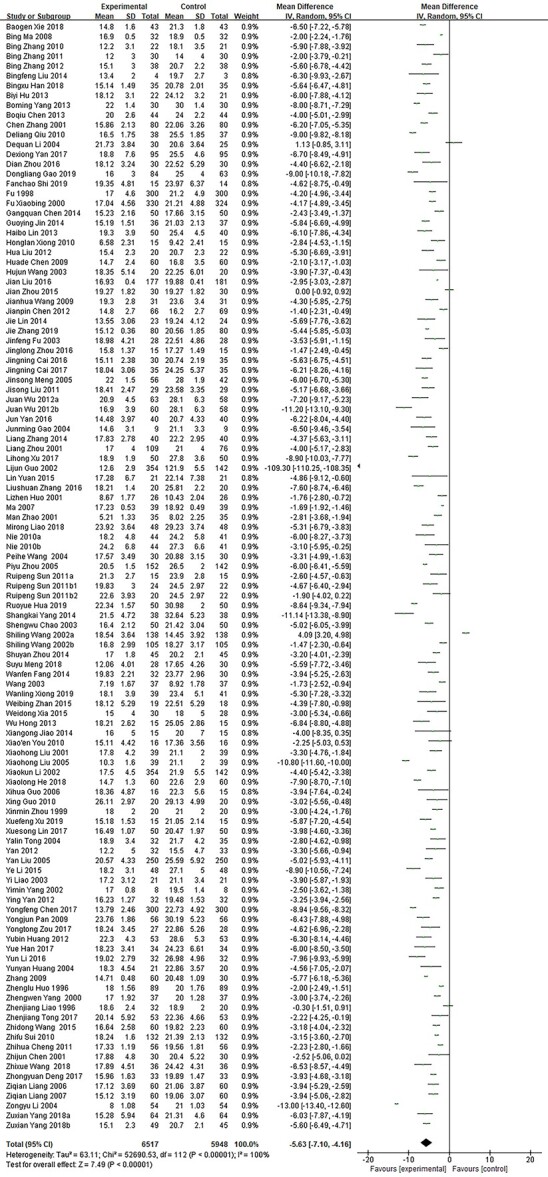
Comparative meta-analysis of the healing time of deep second-degree burn wounds. *CI* confidence interval, *MD* mean difference

A total of 113 studies [10,11,15,16,19–24,28,30–32,34–36,38–53,57,58,61,62,66–69,72–76,78,80–82,84,87–97,100–110,112,115–120,122,123,125–134,136–143,145,146,230,232,233,235,237,238,240,241] enrolling 12 465 cases were conducted to compare the healing time of deep second-degree burn wounds between growth factor and other non-growth factor treatments. The results showed the occurrence of statistical heterogeneity (*p* < 0.00001; *I*^2^ = 100%). Therefore, the random effect model was used for meta-analysis (Figure 3). The results showed that the wound healing time was 5.63 days shorter in the growth factor group than in the control group (MD = −5.63; 95% CI:−7.10 ~ −4.17; *p* < 0.00001).

**Figure 4.**
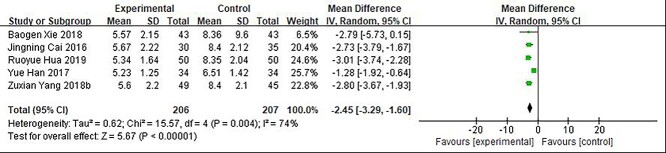
Comparative meta-analysis of the scar score of deep second-degree burn wounds. *CI* confidence interval, *MD* mean difference

### Healing rate comparison of second-degree burn wounds

Healing rate was defined as the proportion of healed wound area compared with the total wound area. Seventeen studies [15,17,20,36,41,42,44,51,52,54,61,68,69,71,72,77,81] enrolling 3184 cases were conducted to compare the healing rate of superficial second-degree burn wounds between growth factor and other non-growth factor treatments. The results showed the presence of statistical heterogeneity (*p* < 0.00001; *I*^2^ = 99%). Therefore, the random effect model was used for meta-analysis (Figure S1, see online supplementary material). The results showed that the average wound healing rate was 15.60% higher in the growth factor group than in the control group (MD = 15.60; 95% CI: 10.51–20.68; *p* < 0.00001). A total of 43 studies [15,20,36,41,42,44,51,52,61,68,69,72,73,81,87,88,91,94,97,99,102,107,108,110,114,117–119,123,124,126,128,129,132,136,138,139,141,143,145,232,233] enrolling 5696 cases served to compare the healing rate of deep second-degree burn wounds between growth factor and other non-growth factor treatments. The results showed the occurrence of statistical heterogeneity (*p* < 0.00001; *I*^2^ = 98%). Hence, the random effect model was used for meta-analysis (Figure S2, see online supplementary material). The results showed that the wound healing rate was 10.84% higher in the growth factor group than in the control group (MD = 10.84; 95% CI: 8.31 ~ 13.37; *p* < 0.00001).

### Infection rate of second-degree burn wounds

Seven studies [16,33,58,76,79,80,82] including 395 cases with superficial second-degree burn wounds compared the infection rate of growth factor and other non-growth factor treatment methods. There turned out to be no statistical heterogeneity between the results (*p* = 0.24; *I*^2^ = 25%). Therefore, the fixed effect model was used for meta-analysis (Figure S3, see online supplementary material). The results showed that the infection rate was lower in the growth factor treatment group than in the non-growth factor group, and the difference was statistically significant (RR = 0.52; 95% CI: 0.39–0.69; *p* < 0.00001). Seventeen studies [16,58,76,80,82,91,94,108,118,119,122,124,128,131,132,135,136] enrolling a total of 1389 patients were conducted to compare the infection rate of deep second-degree burn wounds between growth factor and other non-growth factor treatments. The results showed no statistical heterogeneity (*p* = 0.54; *I*^2^ = 0%). Hence, the fixed effect model was used for meta-analysis (Figure S4, see online supplementary material). The results showed that the infection rate was lower in the growth factor group than in the non-growth factor treatment group (RR = 0.52: 95% CI: 0.42 ~ 0.64; *p* < 0.00001).

### Vancouver scar scale score of deep second-degree burn wounds

Five studies [101,104,108,122,123] including 413 patients compared growth factor with other non-growth factor treatments concerning the deep second-degree burn scar score. The follow-up time was between 6 and 12 months. The results showed the presence of statistical heterogeneity (*p* = 0.004; *I*^2^ = 74%). Therefore, the random effect model was used for meta-analysis (Figure 4). The results showed that the Vancouver scar scale score of the growth factor treatment group was improved as compared with that of the non-growth factor group (5.23 ~ 5.67 *vs* 6.51 ~ 8.4, i.e. 2.45 lower than that of the non-growth factor treatment group) (MD = −2.45; 95% CI: −3.29 ~ −1.6; *p* = 0.004).

### Adverse reactions of deep second-degree burn wounds

Three studies [95,96,124], including 522 patients with deep second-degree burn wounds, compared the incidence of adverse reactions after the treatment with growth factor vs. other non-growth factor treatments. The results showed that no statistical heterogeneity occurred (*p* = 0.29; *I*^2^ = 20%), so the fixed effect model was used for meta-analysis (Figure S5, see online supplementary material). The results showed that the incidence of adverse reactions was lower in the growth factor treatment group than in the non-growth factor group (RR = 0.35; 95% CI: 0.19–0.67; *p* = 0.001).

**Figure 5.**
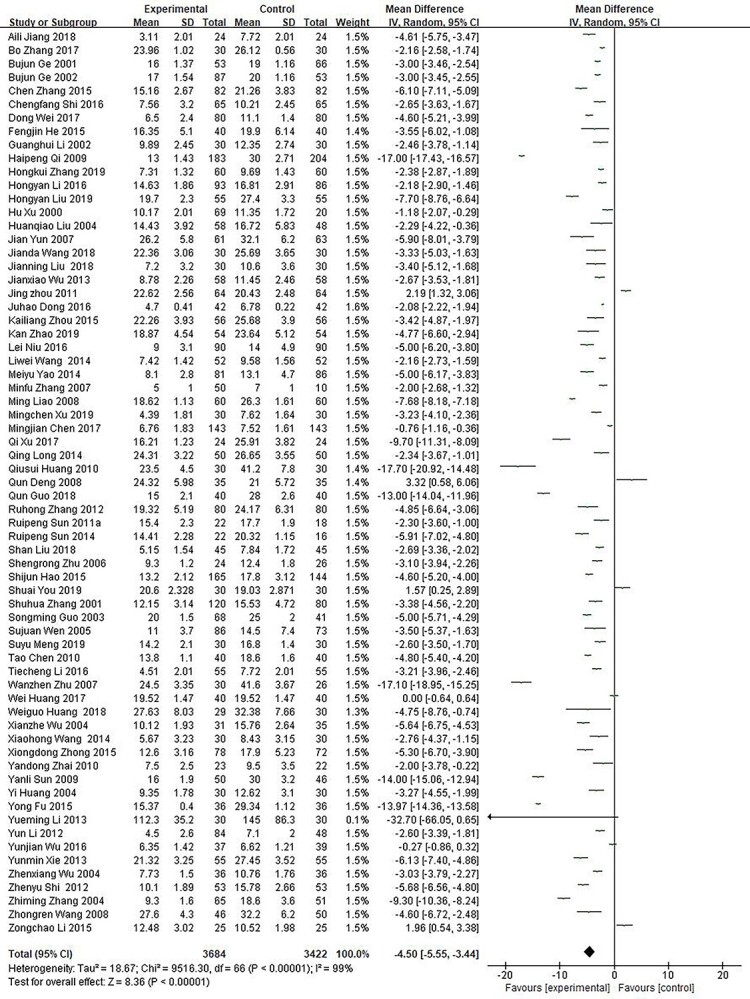
Comparative meta-analysis of the healing time of trauma and surgical wounds. *CI* confidence interval, *MD* mean difference

### Healing time comparison between traumata and surgical wounds

A total of 67 studies [48,147–156,158–164,166–173,175–177,179,181,184–188,190,192–194,196–203,205,206,208–214,216,218–226] including 7106 cases with traumata or surgical wounds served to compare the wound healing time between growth factor and other non-growth factor treatments. The results showed that statistical heterogeneity occurred (*p* < 0.00001; *I*^2^ = 99%). Hence, the random effect model was used for meta-analysis (Figure 5). The results showed that the healing time was 4.50 days shorter in the growth factor group than in the control group (MD = −4.50; 95% CI: −5.55 ~ −3.44; *p* < 0.00001).

### Healing rate comparison of traumata and surgical wounds

Thirteen studies [148,155,165–167,169,170,184,185,191,193,203,228] enrolling 1017 patients with traumata or surgical wounds allowed to compare the rate of wound healing between growth factor and other non-growth factor treatments. The results showed that statistical heterogeneity was present (*p* < 0.00001; *I*^2^ = 99%), so the random effect model was used for meta-analysis (Figure S6, see online supplementary material). The results showed that the wound healing rate in the growth factor group was 7.63% higher than in the control group (MD = 7.63; 95% CI: 4.44 ~ 10.82; *p* < 0.00001).

### Adverse reaction of traumata and surgical wounds

Six studies [157,171,197,215,219,221] including 622 patients with traumata and surgical wounds compared the incidence of adverse reactions after growth factor treatment or other non-growth factor treatment methods. The results were statistically heterogeneous (*p* < 0.0001; *I*^2^ = 84%). Hence, the random effect model was used for meta-analysis (Figure S7, see online supplementary material). The results showed that the incidence of adverse reactions was lower in the growth factor group than in the control group (RR = 0.55; 95% CI: 0.46 ~ 0.65; *p* < 0.00001).

## Discussion

Growth factors are important biologically active molecules which can markedly impact on the wound environment, leading to rapid increases in cell migration, proliferation and differentiation, while regulating the cellular responses inherent to the wound healing process [14]. Recombinant growth factors have been used as adjunctive treatments for acute wounds to accelerate healing, however, the effectiveness and safety of administering these growth factor products under such conditions had not been systematically analyzed. In 2016, Zhang *et al*. [242] performed a meta-analysis concerning growth factor therapy in cases of partial thickness burns. Thirteen studies with a total of 1924 participants were included and the results showed that the topical application of growth factors including FGF, EGF and GM-CSF significantly reduced wound healing time as compared with standard wound care alone. Although these preliminary results seemed to be encouraging, the authors pointed out that high-quality and adequately powered trials were still needed to further confirm their conclusions. Another meta-analysis performed by Abdelhakim *et al*. included 9 clinical studies and has shown that local bFGF treatment accelerated wound healing and prevented pathological scarring. In a similar fashion, the author pointed out that further research was needed to indicate more clinical advantages [243].

In this systematic review, we performed a comprehensive search of relevant clinical studies published in either Chinese or English. We included many studies published in Chinese which had not been considered for evaluation before. Our data show that as compared to non-growth factor treatments, the therapeutic use of growth factor products including FGF, EGF and GM-CSF for acute wounds significantly changed the healing outcome in terms of lessening healing time, heightening healing rate and reducing incidence of infections and adverse reactions. Therefore, our study results positively support the therapeutic use of the current clinically available growth factor products for acute wounds, especially in the case of wounds that tend to have longer healing time.

However, one must point out that out of the 229 studies considered, only 3 were conducted outside China (i.e. in Japan) and reported in English, while the remaining 226 articles, including 7 reported in English and 219 in Chinese, were all carried out within China and reported by Chinese researchers. During the screening period, one randomized clinical trial conducted in the USA showed that epidermal growth factor accelerated skin-graft-donor sites wound healing significantly [9]. However, the types of outcome measurements in this study could not be combined with those from other included studies to conduct meta-analysis. Thus although it was eventually excluded, the results of this study did support our general conclusions. We have to admit that the lack of clinical data from other countries and areas has reduced the evidence’s power level. This is especially true considering that most of the included studies are rated as low-quality ones (Jadad score: 1–2 for 202 papers, 4–5 for 6 papers only). The lack of sufficient clinical data from other countries and areas outside Asia is likely caused by the lack of available growth factor products for treating acute wounds in these places. Becaplermin in Regranex® is the only U.S. Food and Drug Administration (FDA) approved recombinant PDGF product and is only indicated for the treatment of neuropathic ulcers in diabetics. This product carried a boxed warning from the FDA and due to safety issues has been withdrawn in Europe [244]. We were only able to find one study using PDGF gel to treat acute full-thickness punch biopsy wounds on 7 healthy subjects [245]. The results of the study showed PDGF gel was effective in promoting wound healing, which was in accord with the general results of this meta-analysis. Since PDGF has not been officially approved for use on acute wounds, we did not include PDGF in this meta-analysis. However, we believe that when PDGF becomes more widely used for treating acute wounds in the future, it will be meaningful to conduct a more comprehensive evaluation regarding the efficacy and safety issues of all the important growth factor products that are still lacking evidence for clinical use today.

Although this meta-analysis has brought to light encouraging results, the collection of the latter from limited countries and areas (mainly in China) increases the bias of the study. From this standpoint, the evidence supporting the routine therapeutic use of growth factor products for acute wounds is still weak. More high-quality clinical studies and clinical studies from outside of China are needed to further confirm the efficacy, necessity and safety of their clinical application. Despite the possible bias of the conclusions drawn from clinical studies, the current data do show some potential merits of using growth factors to promote acute wound healing. It is interesting to note that several of the included studies focused on the healing of surgical wounds entailing high risks of contamination and infection, such as in the case of perianal surgery [154,214,218,219,223,224,226]. Growth factors were beneficial as they decreased the healing time of such wounds, and therefore decreased the chances of infection and of the development into chronic wounds. Thus, the therapeutic use of growth factors in cases with surgical wounds susceptible to contamination and infection could be a beneficial practice. Again, the need remains for more evidence reported by higher-quality studies.

Moreover, we noted that therapeutically using growth factors for acute wounds not only increased the speed of healing, but also improved the quality of healing in the case of deep wounds. It is well worth pointing out that with growth factors treatments, deep second-degree burn wounds healed with lower scar scores [101,104,108,122,123], which is an important indicator for routine clinical use. It is well known that an increased wound healing time is an important risk factor for hypertrophic scarring in second-degree burns [246]. The current data showed that, instead of causing ‘an overgrowth’, growth factor treatments safely reduced wound healing time by 5.63 days while concurrently decreasing the degree of hypertrophic scarring. Similarly, in their study Abdelhakim *et al*. [243] also pointed out that bFGF might prevent pathological scarring through several cellular mechanisms, such as interfering with myofibroblasts formation and inducing apoptosis. However, longer follow-up times and large-scale clinical trials are still needed to confirm this scar-reducing effect and the causal relationship with reduced wound healing times.

Notably, most of the studies included in this systematic review used only a single growth factor either by itself or combined with other non-growth factor treatments and proved their effectiveness. However, it is yet to be proven that combining different growth factors achieves better clinical results, or whether the contrary is true. Since applying supra-physiological doses of growth factor(s) correlates with an increased risk of cancer, the importance of controlling the spatial–temporal release of growth factors at the wound site and of overcoming this challenge is probably crucial for any successful growth factor-based therapy [244]. Also, as different growth factors partake in the various stages of the wound healing process, using a single growth factor may not suffice for best wound healing. A sophisticated growth factor delivery system enabling a controlled spatial–temporal delivery [13], mimicking the synergistic wound healing activity of the combined release profiles of growth factors in real physiological situations, could be a promising direction for future research. Currently, the use of platelet rich plasma (PRP) to promote refractory wound healing has already supplied a hint for applying growth factor compounds in a more effective fashion. However, PRP has not been routinely used on acute wounds due to economic considerations. More in-depth study of the PRP’s spatial–temporal working mechanism might provide stronger evidence to develop recombinant growth factor combination products for promoting acute wound healing in the future.

## Conclusions

With the systematic review and evaluation of the currently available evidence, we conclude that the therapeutic use of growth factors including EGF, FGF and GM-CSF is effective and safe in the treatment of acute skin wounds, especially in the case of wounds entailing higher risks of infection. However, the need still remains for more higher-quality studies to further strengthen our conclusion.

## Supplementary Material

Supplementary_data_tkac002

## Data Availability

Data are available from PubMed/Medline, Cochrane Library, Cochrane CENTRAL, ClinicalTrials.gov, Chinese Journal Full-text Database (CNKI), China Biomedy Medicine disc (CBM), Chinese Scientific Journal Database (VIP), and Wanfang Database (WFDATA).

## Abbreviations

CI; Confidence interval; EGF; Epidermal growth factor; FGF, Fibroblast growth factor; GM-CSF: Granulocyte-macrophage colony stimulating factor; MD, Mean difference; PDGF: Platelet-derived growth factor; PRP: Platelet rich plasma; rbFGF: Recombinant bovine basic fibroblast growth factor; rh-aFGF: Recombinant human acidic fibroblast growth factor; RR: Relative risk.

## References

[1] Cañedo-Dorantes L , Cañedo-Ayala M. Skin acute wound healing: a comprehensive review. Int J Inflam. 2019;2019:3706315. 31275545 10.1155/2019/3706315 PMC6582859

[2] Rodrigues M , Kosaric N, Bonham CA, Gurtner GC. Wound healing: a cellular perspective. Physiol Rev. 2019;99:665–706. 30475656 10.1152/physrev.00067.2017 PMC6442927

[3] Nour S , Imani R, Chaudhry GR, Sharifi AM. Skin wound healing assisted by angiogenic targeted tissue engineering: a comprehensive review of bioengineered approaches. J Biomed Mater Res Part A. 2021;109:453–78. 10.1002/jbm.a.37105 32985051

[4] Li S , Liu Y, Huang Z, Kou Y, Hu A. Efficacy and safety of nano-silver dressings combined with recombinant human epidermal growth factor for deep second-degree burns: a meta-analysis. Burns. 2021;47:643–53. 31982184 10.1016/j.burns.2019.12.015

[5] Lin XY , Wang H, Tan Y. Role of hepatocyte growth factor in wound repair. Acta Zhongguo Yi Xue Ke Xue Yuan Xue Bao. 2018;40:822–6. 10.3881/j.issn.1000-503X.10246 30606395

[6] Brem H , Howell R, Criscitelli T, Senderowicz A, Siegart N, Gorenstein S, et al. Practical application of granulocyte-macrophage Colony-stimulating factor (GM-CSF) in patients with wounds. Surg Technol Int. 2018;32:61–6. 29611156

[7] Frati C , Scarpa C. Treatment of experimental mouse burns with E.G.F. (epidermal growth factor) applied locally as a lotion. G Ital Dermatol Minerva Dermatol. 1971;46:73–6. 5108745

[8] Benington L , Rajan G, Locher C, Lim LY. Fibroblast growth factor 2-a review of stabilisation approaches for clinical applications. Pharmaceutics. 2020;12:508. 10.3390/pharmaceutics12060508 PMC7356611 32498439

[9] Brown GL , Nanney LB, Griffen J, Cramer AB, Yancey JM, Curtsinger LJ, 3rd, et al. Enhancement of wound healing by topical treatment with epidermal growth factor. N Engl J Med. 1989;321:76–9. 2659995 10.1056/NEJM198907133210203

[10] Fu X , Shen Z, Chen Y, Xie J, Guo Z, Zhang M, et al. Randomised placebo-controlled trial of use of topical recombinant bovine basic fibroblast growth factor for second-degree burns. Lancet. 1998;352:1661–4. 9853438 10.1016/S0140-6736(98)01260-4

[11] Ma B , Cheng DS, Xia ZF, Beng DF, Lu W, Cao ZF, et al. Randomized, multicenter, double-blind, and placebo-controlled trial using topical recombinant human acidic fibroblast growth factor for deep partial-thickness burns and skin graft donor site. Wound Repair Regen. 2007;15:795–9. 18028126 10.1111/j.1524-475X.2007.00307.x

[12] Yamakawa S , Hayashida K. Advances in surgical applications of growth factors for wound healing. Burns & trauma. 2019;7:10. 30993143 10.1186/s41038-019-0148-1 PMC6450003

[13] Park JW , Hwang SR, Yoon IS. Advanced growth factor delivery Systems in Wound Management and Skin Regeneration. Molecules. 2017;22:1259. 10.3390/molecules22081259 PMC6152378 28749427

[14] Moher D , Shamseer L, Clarke M, Ghersi D, Liberati A, Petticrew M, et al. Preferred reporting items for systematic review and meta-analysis protocols (PRISMA-P) 2015 statement. Syst Rev. 2015;4:1. 25554246 10.1186/2046-4053-4-1 PMC4320440

[15] Pan YJ , Zong YX, Bai SP, Zhang SZ, Yang H. Repair of 58 cases of diabetic burn wounds. Jiangxi Medical Journal. 2009;44:1089–90.

[16] Hong W , Li SF, Gan ZL. BFGF combined compound sulfadiazine zinc Pingment gel with Vaseline gauze therapy for II burn wound in children. China Medicine And Pharmacy. 2013;3:112–38.

[17] Guo Y , Wang SC. Clinica study of Er Huang ointment combined with recombinant human granulocyte macrophage stimulating factor gel in the treatment of superficial II degree burns. Zhong Yi Yao Dao Bao. 2017;23:90–2.

[18] Ma JB . Effect of vacuum sealing drainage combined with recombinant bovine basic fibroblast growth factor on electric burn wound of extremities. Chin J Crit Care Med (Electronic Edition). 2014;7:126–8.

[19] Huang YY , Qin QH, Bian JM. Effect of sulfadiazine silver cream combined with recombinant human epidermal growth factor on burn wounds in children. Guang Xi Yi Ke Da Xue Xue Bao. 2004;21:895–6.

[20] Li XK , Hong A, Xu H, Yao CC, F XB, Lin J. The clinical study of recombinant bovine basic fibroblast growth factor on wounds healing. Journal of Jinan University (Medicine Edition). 2002;23:22–7.

[21] Chen ZJ , Wang Y, Wang ZH. Application of basic fibroblast growth factor bFGF in small area burn wound healing. Tianjin Yao Xue. 2001;13:46–8.

[22] Gao JM , Chen CH, Kou JB, Zhang LS, Li GC, Fan YN, et al. Effect of basic fibroblast growth factor on wound healing. Shanxi Med J. 2004;33:967–8.

[23] Huo ZL , Ge SD, Chen WL, Zhao XL, Mu XX, Liu SK. Application of basic fibroblast growth factor in burn wound. Acad J Second Mil Med Uni. 1996;S1:94–6.

[24] Li ZY , Huang LB, Yang XB, Wang CG. Application of basic fibroblast growth factor in burn wound. Acad J Second Mil Med Uni. 2004;25:3.

[25] Hu XM , Ma J, Zhang YF, Wang WB. Effect of Jinfuning combined with silver sulfadiazine cream in the treatment of children with second degree scald burns. Medical Journal of GEHQ. 2012;14:220–1.

[26] Gong YJ . Effect of Genetime on small area superficial second degree burn. Modern Nursing. 2007;13:732.

[27] Luo HX . Study on The Application of Chitosan in Children’s Superficial Second-Degree Burn Wounds. Chongqing Medical University, 2014.

[28] Liao ZJ , Huang BG, Xiang J, Xu WS. Clinical evaluation of epidermal growth factor in human burn wounds. Acta Universitatis Medicinalis Secondae. 1996;16:266–8.

[29] Guo Y , Yu JJ, Lv GZ. The clinical observation of applying recombinant human epidermal growth factor to treat superficial second-degree burn. Chin J Injury Repair and Wound Healing. 2009;4:683–7.

[30] Liu XH , Tang MR, Zhang SD, He J. The clinical study of recombinant human basic fibroblast growth factor stimulating the wound surface healing. J Chin Med Univ. 2001;30:38–9.

[31] Liu H , Tao HJ. A study of the treatment of 67 cases of face and neck burns in children. Journal of Clinical and Experimental Medicine. 2012;11:31–2.

[32] Gao DL , Gao DD, Xue HB, Yang XM, Bai C. Application of in situ regeneration combined with rh-EGF in the treatment of facial burn patients. Xin Li Yue Kan. 2019;17:21–2.

[33] Li HX . Effect of recombinant epidermal growth factor on wound healing of second degree burn. International Medicine and Health Guidance News. 2003;9:33–4.

[34] Liu Y , Fu YX. Healing effect of bFGF on burn wound degree II of children. J Pediatr Pharm. 2005;11:20–1.

[35] Lin J , Dai ZQ. Clinical observation of recombinant bovine basic fibroblast growth factor gel for treatment mild burns. Chinese Journal of Medicinal Guide. 2014;16:1016–7.

[36] Guo LJ , Li XK, Xu H, Yao CC. Curative effect of recombinant bovine basic fibroblast growth factor on burn wound degree II. Chin J Biologicals. 2002;15:310–1.

[37] Fan GC , Rong XZ, Zhang T, Li SZ. Clinical observation of recombinant bovine basic fibroblast growth factor in the treatment of burn wounds. Guang Zhou Yi Yao. 2018;49:25–39.

[38] Meng SY , Zhao P, Ma YN, Xu WH. Effect of recombinant bovine basic fibroblast growth factor on burn wound. J Med Theor & Prac. 2018;31:539–40.

[39] Guo X , Tan MY, Guo L, Xiong AB, Li YG, He XC. Clinical study on repair of burn wounds of degree II with recombinant human epidermal growth factor in elderly patients. Chinese Journal of Reparative and Reconstructive Surgery. 2010;24:462–4. 20459012

[40] Fang WF , Li HL. Clinical observation of recombinant human epidermal growth factor in promoting wound healing of second degree burn. Journal of China Prescription Drug. 2014;12:25–6.

[41] Liang ZQ , Li HM, Meng CY. Repair of second degree facial burns in children using recombinant human epidermal growth factor. Journal of Clinical Rehabilitative Tissue Engineering Research. 2007;11:1974–5.

[42] Liang ZQ , Li HM, Meng CY, Sun XF. A comparison of repaired effect of recombinant human epidermal growth factor for the facial degree II burn wounds. Chinese Journal of New Drugs. 2006;15:812–4.

[43] Huo LZ , Liu XL, Huang J, Li XJ, Zhong SH, Liang DR. Effect of recombinant human epidermal growth factor in treatment of burn wound and donor site. Academic Journal of Guangdong College of Pharmacy. 2001;17:60–1.

[44] Fu JF , Chen B, Zhang J, Liang M, Cao WD, Wei DN, et al. Clinical observation of recombinant human epidermal growth factor in the treatment of burn wounds. J Trauma Surg. 2003;5:385.

[45] Liao Y , Guo L, Ding EY, He XC, Xie XQ, Xia DL. A comparative study on burn wound healing treated by different methods of recombinant human epidermal growth factor. Chinese J Reparative and Reconstructive Surgery. 2003;17:301–2. 12920719

[46] Li DQ , Li JB, Liang MH. The negative result of recombinant human epidermal growth factor effecting on wounds. Yi Xue Wen Xuan. 2004;23:133–4.

[47] Liu XH , Jiang N, Tan C, Tang MR. The clinical study of recombinant human basic fibroblast growth factor in stimulating the wound surface healing. Acta Academiae Medicinae Jiangxi. 2005;45:92–4.

[48] Chao SW , Li Y, Wang XZ. Application of recombinant human basic fibroblast factor in burn wound. China Journal of Modern Medicine. 2003;13:52–3.

[49] Guo XH . Clinical observation on recombinant human basic fibroblastic growth factor treating II burn. Mod Diagn Treat. 2006;17:215–6.

[50] Liu BF . Recombinant human basic fibroblast growth factor in treating burns and other skin injuries. Mod Diagn Treat. 2014;25:5535–7.

[51] Chen GQ . Clinical study of recombinant human acidic fibroblast growth factor in burn treatment. Lin Chuang Yan Jiu. 2014;11:47–8.

[52] Sun RP , Zhao LK, Sun J. Clinical observation of rh-aFGF in the treatment of second degree burn. Hebei Medical Journal. 2011;33:2293–4.

[53] Qiu DL , Lin YH, Li CG. Clinical research of bFGF combined with Bashigao in treating II burned wound. Hebei Medicine 2010; 16:331–3.

[54] Sun RP , Zhao LK, Sun J, Li DJ, Huai Q, Xu LJ. Clinical observation of modified chitin combined with recombinant human basic fibroblast growth factor in treating Superfical partial-thickness burn wound. Chin J Injury Repair and Wound Healing. 2018;13:269–72.

[55] Tan JT , Zhang B, Wei P, Yu LY. Effect of basic fibroblast growth factor on burn wound healing. China Journal of Modern Medicine. 2000;10:10–1.

[56] Song XN , Jing HP, Zou YH. Effect of basic fibroblast growth factor on 50 cases of burn wounds. Zong Zhuang Bei Bu Yi Xue Xue Bao. 2003;5:32–3.

[57] Tong YLT , Zhu JH, Miao HC, Pan K, Yang FW, Kong ZB, et al. A clinical observation of Kangfushuang to treat the partial and full thickness burn wounds. Chinese Journal of Rehabilitation Medicine. 2004;19:3.

[58] Shi FC . Clinical analysis of Nano silver dressing combined with recombinant human epidermal growth factor in the treatment of second degree burn. J Huaihai Med. 2019;37:177–9.

[59] Sun RP , Zhao LK, Sun J, Xu LJ, Zheng WL, Li DJ. Effect of external application of lyophilized recombinant human acidic fibroblast growth shadow in the treatment of superficial second degree burn. Zhong Guo Xiang Cun Yi Yao Za Zhi. 2015;22:11–2.

[60] Tan JT , Zhang B, Yu LY, Li W. Effect of recombinant human epidermal growth factor on wound healing of second degree burn. Shi Yong Yi Xue Za Zhi. 2001;17:872–3.

[61] Wang PH , Qi SZ, Peng YZ. Comparison of the wound healing Acceleraton of recombinant human epidermal growth factor and recombinant human fibroblast growth factor in the treatment of burn wounds. Clin J Med Offic. 2004;32:35–7.

[62] Yang ZW , Yang X, Zhou M. Basic fibroblast growth factor promotes burn wound healing. Modern Rehabilitation. 2000;4:73.

[63] Wang XH , Gu CZ, Chen ZY, Li HJ, Li JX. Clinical observation of basic fibroblast growth factor on promoting burn wound healing. Xin Jiang Yi Xue. 2000;30:27.

[64] Ye WG , Xue RH, Ye PG, Zhou B. Observation on the effect of combined application of Genetime and silver sulfadiazine in dressing change of second degree burn wound. J Med Theor & Prac. 2008;21:1189–90.

[65] Wang JJ , Hu QX, Liu Q. Clinical observation of Genetime combined with Nano silver dressing in the treatment of second degree burn wound. Inner Mongolia Med J. 2010;42:830–1.

[66] Xiong HL , Zhong XL, Fu XH. Clinical study of Genetime combined with amniotic membrane covering in the treatment of burn wounds. Hu Shi Jin Xiu Za Zhi. 2010;25:349–50.

[67] Wang JH , Xu GS, Wang Y, Zhu ZJ. Clinical observation of the effects of Trolamine cream on burn wounds. Acta Acadmiae Medicinae Qindao Universitatis. 2009;45:5.

[68] Xiong WL . Comparative analysis of external application of recombinant bovine basic fibroblast growth factor and silver sulfadiazine in the treatment of burn wounds. Guide of China Medicine. 2019;17:154–5.

[69] Wang SL , Chai JK, Shen ZY, Zhou YP, Liao ZJ, Zhou L, et al. Phase IV Multicenter clinical study of recombinant human epidermal growth factor derivative. Chinese Critical Care Medicine. 2002;14:5.

[70] Xu FR , He MW, Yang F. The clinical effect of recombinant bovine basic fibroblast grow promote wound healing after burn. Chin J of Clinical Rational Drug Use. 2016;9:35–8.

[71] Xiong WL . Efficacy of recombinant human epidermal growth factor gel in children with superficial second degree burn wounds. Guide of China Medicine. 2018;16:138.

[72] Yang YM , Zhang ZX, Sun YW. Effect of recombinant human basic fibroblast growth factor on the repair of burn wound. Xi Bei Yao Xue Za Zhi. 2002;17:26.

[73] Wang HJ , Qi SZ, Yang JM, Li XD. Clinical observation of rh-bFGF in treatment of II burns in man. China Pharmacy. 2003;14:480–1.

[74] Zhou XM , Wang XM. Obseruation of bFGF on burned wound. Hebei Medicine. 1999;5:29–30.

[75] Zhou PY , Yang XM. Effect of bFGF on treatment of 152 cases of second degree burn wound. Zhong Guo She Qu Yi Shi. 2005;7:41.

[76] Zhan WB . Efficacy of recombinant human epidermal growth factor combined with Nano-siver dressings on second-degree burns. Chinese Journal of General Practice. 2015;13:926–8.

[77] Zhang BL , Zhang N, Zhang T. Curative effect analysis of recombinant bovine basic fibroblast growth factor gel on burn wound. Chinese Community Doctors. 2014;30:58–9.

[78] Zhang C , Hong SW, Gu C, Huang ZX, Du SL. Clinical observation of recombinant bovine basic fibroblast growth factor in the treatment of second degree burn wounds. Chin J Burns. 2001;17:246.

[79] Zhao PD , Liu YL. Effect of recombinant human epidermal growth factor on second degree burn wound healing. International Medicine and Health Guidance News. 2015;21:2819–21.

[80] Zou YT , Lian ZP, Lin S, Zhang BQ, Dai SG. Effect of recombinant human epidermal growth factor combined with Nano silver dressing on second degree burn. Practical Clinical Medicine. 2017;18:53–4.

[81] Zhou L , Wang SL, Ma JL, Chai JK, Li LG. A Multicenter study of recombinant human epidermal growth factor for topical treatment of burn wounds. Chin J New Drugs Clin Rem. 2001;20:337–40.

[82] Zhang B . Recombinant human epidermal growth factor in treatment of second degree burn wounds. Med J West China. 2012;24:561–2.

[83] Zheng ZZ , Liu JF, Xie WG, Wu RZ, Zhou HP, Wan SY. Applicaton of recombinant human epidermal growth factor in the treatment of children with second degree burn. J Huazhong Univ Sci Tech. 2003;32:667–8.

[84] Zhou SY , Li H, Wang JH, Liu XY, Qi CC, Zhang MZ. Application of recombinant human acidic fibroblast growth factor in burn treatment. Shanxi Med J. 2014;43:185–6.

[85] Wu XY , Qin XQ. Observation on the effect of bFGF combined with Comfeel hydrocolloid gauze in the treatment of mild to moderate burns in infants. Int J Nurs. 2015;34:2430–2.

[86] Lu DP . Application of basic fibroblast growth factor in Pediatric burn wound. Acta Medicinae Sinica. 2002;15:43–4.

[87] Hu BY . Clinical research of BFGF and far infrared ray to promote Adustum. Chinese Journal of General Practice. 2013;11:1565–6.

[88] Huang YB , Chen WB, Hu J, Su YS. Clinical study of bFGF combined with local oxygen therapy in promoting deep second degree burn wound healing. Asia-Pacific Traditonal Medicine. 2012;8:147–8.

[89] Liu JS , Fang Y, Yao M, Yu WR, Li XG. Effect of recombinant human granulocyte-macrophage Colony-stimulating factor on wound debridement and healing of deep II thickness burn. Chinese Journal of Reparative and Reconstructive Surgery. 2011;25:1059–62. 21991809

[90] He XL , Zhang B, Li W, Li Z, Chen B. Clinical effect of epidermal growth factor combined with Polymyxin B for wound scar in elderly patients with deep second-degree burns. Pract Geriatr. 2018;32:828–30.

[91] Cheng ZH , Peng XP, Peng WF, Zhou PY. Clinical observation of Fulin honey combined with topical recombinant human granulocyte macrophage stimulating factor gel in the treatment of deep second degree burns. Chin J Injury Repair and Wound Healing (Electronic Edition). 2011;6:259–63.

[92] Chen BQ , Peng WY, Yu JC, Qiu JC, Liu BF, Zhao WF. Research on the therapeutic effect of epidermal growth factor gel and collagen dressing in the treatment of facial deep II degree burn. Lin Chuang Yi Xue Gong Cheng. 2013;20:1127–8.

[93] Chen JP , Wen SH, Li Z. Combined Administration of Moist Exposed Burn Ointment (MEBO) and basic fibroblast growth factor (bFGF) in children deep II degree burn wounds. Lin Chuang Yi Xue Gong Cheng. 2012;19:1134–5.

[94] Liao MR , Wang HL, Guo ZX. Effect of Nano-silver dressing combined with recombinant bovine basic fibroblast growth factor on the expression of inflammatory factors, EGF and VEGF in deep second degree burn wounds. China Modern Doctor. 2018;56:89–96.

[95] Li Y , Jiao JQ, Huang Z, Hu WG. Safety and effectiveness of Nano silver dressing combined with recombinant human epidermal growth factor gel on patients with deep II degree burn wounds. Chinese Journal of Medicinal Guide. 2015;17:941–2.

[96] Han BX . Effect of recombinant human basic fibroblast growth factor on deep second degree burn. Chin J of Clinical Rational Drug Use. 2018;11:60–1.

[97] Lin XS , Wang L, Liu SJ, Cai YN. Clinical observation of recombinant human granulocyte/macrophage Colony stimulating factor hydrogel for topical application in treating burn wounds. Modern Practical Medicine. 2017;29:516–8.

[98] Zeng JD . Clinical Study on Treating Deep Second Degree Burn Wounds with Recombinant Human Granulocyte-Macrophage Colony-Stimulating Factor Hydrogel. Luzhou Medical College, 2012.

[99] Li L . Effect of insulin combined with recombinant human acidic fibroblast growth factor on deep second degree burn wound healing. Shan Dong Yi Yao. 2014;54:75–6.

[100] Meng JS , Li CM, Xu LF, Wang J, Zhang K, He ZY. Effect of growth factor on deep second degree burn wound. Ren Min Jun Yi. 2005;48:570–1.

[101] Han Y , Ren J, Wu JH, Wang Y. Effect of recombinant human epidermal growth factor combined with sulfadiazine zinc gel on deep second degree burn wounds. Journal of Guangxi Medical University. 2017;34:1354–7.

[102] Chen YF , Shi HZ. Effect observation of rh EGF combined with mupirocin in the treatment of deep second burn wound. Lin Chuang Yi Xue. 2017;93–4.

[103] Li Y . Clinical analysis of recombinant human epidermal growth factor gel combined with Nano silver antibacterial gel in treatment of deep second degree burn wounds. Henan Journal of Surgery. 2016;22:59–60.

[104] Hua RY . Application of rhEGF in the process of facial deep second degree burn wound repair. Chinese Journal of Aesthetic Medicine. 2019;28:36–8.

[105] Jin GY , Fan YF, Chen C, Zhang C, Wu TB. Effect of recombinant human basic fibroblast growth factor on deep second degree burn wound healing. Modern Practical Medicine. 2014;26:480–508.

[106] Cai JN , Sun YJ, Xie XF, Li B, Zou XF. The effect analysis of wound dissolution on deep second degree burn by rhGM-CSF. Zhong Guo Lin Chuang Yi Sheng Za Zhi. 2017;45:39–42.

[107] Lin HB . Effect of recombinant human granulocyte macrophage Colony stimulating factor gel on burn wounds. Contemporary Medicine. 2013;19:46–8.

[108] Cai JN , Li B, Xie XF, Zou XF, Wu SJ, Li BL. Effect of recombinant human acidic fibroblast growth factor on deep second degree burn. Zhong Guo Lin Chuang Yi Sheng Za Zhi. 2016;44:69–71.

[109] Sui ZF , Gu TM, Yang RY, Zhao ZL, Gu Y. Clinical observation of Gaifu in treating deep burn wounds. Chinese Journal of Aesthetic Medicine. 2010;19:753–4.

[110] Tong ZJ , Li Y. Clinical observation on therapeutic effects of combined recombinant human epidermal growth factor (Rh-EGF) gel and sulfadiazine zinc silver ointment in the treatment of deep II degree burn wounds. Asia-Pacific Traditonal Medicine. 2017;13:3.

[111] Song ML , Yang CB, Li CL, Luo GC, He XD, Xiao Y, et al. Clinical effect of recombinant bovine basic fibroblast growth factor gel in Asssisting wound Repir for deep second degree burn wound. China Prac Med. 2018;13:139–40.

[112] Sun RP , Zhao LK, Sun J, Ma JY, Li DJ, Zheng WL, et al. Effect of recombinant human aFGF on deep second degree burn. Chinese Journal of Reparative and Reconstructive Surgery. 2011;25:639–40.

[113] Qu KP . Effects of Recombinant Human Granulocyte-Macrophage Colony-Stimulating Factor Hydrogel on Healing of Deep Partial-Thickness Burn Wounds and Its Mechanism Analysis. Qingdao University, 2017.

[114] Wang H . Study of Recombinant Human Granulocyte Macrophage Colony-stimulating Factor on the Healing of Deep Partial Thickness Facial Burns in Pediatric Patients. Jilin University, 2014.

[115] Xu XF . Effect of rh-bFGF on deep second degree burn wound healing. Shenzhen Journal of Integrated Traditional Chinese and Western Medicine. 2019;29:195–6.

[116] Wang ZX , Yu Q, Xiao JZ. Comparative analysis between rhGM-CSF gel and acellular xenografts dermis on wound healing effects in patients with deep second degree burn. Med & Pharm J Chin PLA. 2018;30:54–71.

[117] Xu LH . Effect of VSD technique combined with epidermal growth factor solution on wound healing and inflammatory response in patients with deep burn. Chinese Journal of Aesthetic Medicine. 2017;26:4.

[118] Yan Y , Huang GY, Wang HW, Chen G, Ding WX, Zhou PY. Observation of clinical efficacy of Ai Kang Fu ag+ dressings combined with recombinant human epidermal growth factor gel to treat deep II burn residual wounds. China Medical Herald. 2012;9:35–7.

[119] Yang ZX , Li T, Xu JC. Therapeutic effect of mupirocin ointment combined with recombinant human gametocyte/macrophage Colony stimulating factor gel in external use on deep second degree burns. Hebei Medical Journal. 2018;40:1845–8.

[120] Yang SK . Relationship between wound treatment and healing of deep second degree burn. Chinese and Foreign Medical Research. 2014;12:132–3.

[121] Wen CQ , Zhao XZ, Zhang GA. Clinical curative effect observation of recombinant human granulocyte-macrophage Colony-stimulating factor gel on wound healing in patients with deep partial thickness burns. Chin J Injury Repair and Wound Healing. 2016;11:215–8.

[122] Yang ZX , Li T, Xu JC. Effect of rh-aFGF on deep second degree burn after Escharectomy. Ji Lin Yi Xue. 2018;39:697–9.

[123] Xie BG , Huang YX, Chen J, Xu ZX. Evaluation of the efficacy of rh-aFGF in the treatment of second degree deep burns after tangential excision. China Medical Cosmetology. 2018;8:54–7.

[124] Wang L . Recombinant bovine basic fibroblast growth factor gel combined with Nano silver dressing for promoting wound healing in 78 cases. Clin Med. 2015;24:2.

[125] Wang ZD . Observation on effect of recombinant bovine basic fibroblast growth factor (rb-bFGF) gel for treating Pediatric mild to moderate deep II degree burn wounds. China & Foreign Medical Treatment. 2015;15:3.

[126] You XE , Deng JY, Zhu XF. Clinical observation of recombinant human epidermal growth factor gel in treating deep second degree wounds. Hai Xia Yao Xue. 2010;22:170–1.

[127] Yang BM . Clinical observation of recombinant human epidermal growth factor in the acceleration of deep II degree burn wound healing. China Modern Medicine. 2013;20:99–100.

[128] Zhang L , Guo JL, Zhu CL, Gu ZQ. Clinical study of rb-bFGF combined with Nano silver dressing in the treatment of non-functional deep second degree burn wounds in children. Qingdao Med J. 2014;46:433–4.

[129] Zhou J , Li YX, Chi YF. Observation on effect of vacuum-assisted closure treatment combined with flushing with epidermal growth factor solution in treating deep II degree burn wound. Infect Inflamm Rep. 2015;16:49–51.

[130] Zhang B , Wei SQ, Xu H, He HM, Yang WB, Wei YF, et al. Clinical observation of sulfadiazine silver cold cream mask containing recombinant human epidermal growth factor in treating 22 cases of deep II degree burn of the face. Guangxi Medical Journal. 2010;32:561–3.

[131] Zhang B , Xu H. Clinical observation on the healing of facial deep II degree burn wounds with recombinant human epidermal growth factor mask. Chin J Injury Repair and Wound Healing (Electronic Edition). 2011;6:3.

[132] Zhou JL , Guo JL, Jin XM, Zhang TJ. The clinical research of combined application of Nano silver antimicrobial dressing with Rb-bFGF in children with deep II degree burn wound. Acta Acad Med Weifang. 2016;38:394–6.

[133] Zhao M , Zheng Y. Application of recombinant basic fibroblast growth factor in deep burn injury. Modern Rehabilitation. 2001;5:94.

[134] Zhang J . Therapeutic effect of recombinant human granulocyte macrophage Colony-stimulating factor gel on deep II degree burn wounds. Ji Cen Yi Xue Lun Tan. 2019;23.

[135] Zhang J , Zhang XZ, Li H. Clinical observation of recombinant human epidermal growth factor in promoting deep second degree burn wound healing. Chinese Primary Health Care. 2010;24:127–8.

[136] Zhang LS , Tian P. Comparison of recombinant human granulocyte-macrophage Colony stimulating factor gel and acellular skin of treating deep second degree burn wound in clinical effect. Journal of Clinical and Experimental Medicine. 2016;15:662–4.

[137] Deng ZY . Clinical observation on the treatment of deep second degree burn with rhGM-CSF and silver sulfadiazine. J Clin Res. 2017;34:932–4.

[138] Chen HD , Bian HN, Zheng SY, Gao H, Xiong B, Liu ZA, et al. Combined use of Fulin honey and recombinant human epidermal growth factor gel for treatment of deep II degree burn of the face. Chin J Traumatol. 2009;25:2.

[139] Liu J , Liao ZJ, Zhang Q. Phase IV clinical trial for external use of recombinant human granulocyte-macrophage Colony-stimulating factor gel in treating deep partial-thickness burn wounds. Chin J Burns. 2016;32:542–8. 10.3760/cma.j.issn.1009-2587.2016.09.007 27647071

[140] Yan J , Wang HZ, Wang P. Efficacy of recombinant human epidermal growth factor gel in the treatment of deep second degree burn wounds. Chinese Journal of Trauma and Disability Medicine. 2016;24:173–4.

[141] Jiao XG , Li H, Jiang ZJ, Yang L, Zhou JM, Li F, et al. Effect of recombinant human granulocyte macrophage Colony stimulating factor combined with silver sulfadiazine on deep second degree burn wound caused by nitrate fire. Chin J Burns. 2014;30:367–9.

[142] Xia WD , Wan L, Yang RJ, Ling XW, Lin C. Comparison of clinical effects of recombinant human granulocyte Colony stimulating factor gel and xenograft on deep second degree burn wounds. Chin J Burns. 2015;31:216–7.

[143] Ma B , Zhu SH, Cheng DS, Xiao SC, Wang GY, Ben DF, et al. Clinical observation of recombinant human acidic fibroblast growth factor in the treatment of deep second degree burn wounds. Chin J Burns. 2008;24:223.

[144] Shi C , Wang DK, Geng ZH. Clinical study of Dragon's blood combined with recombinant bovine basic fibroblast growth factor gel in treatment of burns. Journal of Hebei Medical University. 2018;39:1217–20.

[145] Wu J , He QF, Lin L. Basic fibroblast growth factor combined with heparin therapy and red light therapy in enhancing burn wound healing. Chinese Journal of Rehabilitation. 2012;27:15–7.

[146] Zhou D , Lv GZ. Comparative study of rhGM-CSF and Nano silver on deep second degree scald wound healing. Hebei Medicine. 2016;22:776–8.

[147] Ge BJ , Guo SM. Clinical application of rbFGF in wound healing after Hemorrhoids and fistulas operation. Shang Xi Yi Xue Za Zhi. 2001;30:296–7.

[148] Niu L , Liu J, Meng LJ. Effect of rh-aFGF in the treatment of facial wounds after high-frequency electro-ionizaiton therapy. Journal of Hebei United University (Health Sciences). 2016;18:216–9.

[149] Dong JH . Observation on the effect of bFGF combined with mupirocin in the treatment of skin abrasion. China’s Rural Health. 2016;20:67–9.

[150] Chen MJ , Xia L, Zhou ZG. Comparison of the effect of epidermal growth factor and infrared irradiation on wound healing. Zhe Jiang Lin Chuang Yi Xue. 2017;19:688–9.

[151] Hao SJ , Sun XJ, Yuan B, Zhang Y, Ying YL, Huang J. Clinical observation of compound Schizonepeta fumigation lotion combined with recombinant human basic fibroblast growth factor in the treatment of 309 cases after anal fissure operation. Jie Zhi Chang Gang Men Wai Ke. 2015;21:72–3.

[152] Liu HQ , You MH. Effect of dressing change of bFGF on wound healing after anal operation. J Nurs Sci. 2004;19:51–2.

[153] Li YM , Tao PY, Huang CQ, Ning Z, Yang XL, Fan PL, et al. Effect observation and nursing of sulfadiazine silver cream in the treatment of skin necrosis caused by Chinese cobra bite. Guangxi Medical Journal. 2013;35:934–6.

[154] Songmin G , Bujun G. Effect of basic fibroblast growth factor on wound healing after perianal operation. Fudan Univ J Med Sci. 2003;30:74–6.

[155] Huang QS , Hou XH, Hu Y. Clinical effect of basic fibroblast growth factor on the healing of the cervical wound of Cervial intraepithelial neoplasia after loop electrosurgical excision procedure. Strait Pharmaceutical Journal. 2010;22:115–7.

[156] Chen T , Chen ZP, Ding ZL. The BFGF effect on the bad injured skin area after Bromhidrosis surgery. Nei Meng Gu Zhong Yi Yao. 2010;7:36–7.

[157] Li M , Li XL, Li XY, Zhang SM, Yu HQ, Deng LN, et al. Clinical effectiveness of the united application with rbFGF and ACRSC on postoperative wound-healing in treatment of acne scar using fractional erbium laser MCL30. Chin J Derm Venereol. 2015;29:1100–2.

[158] Ge BJ , Guo SM. Curative effects of basic fibroblast growth factor on anus wound healing. Chinese J Reparative and Reconstructive Surgery. 2002;16:345–7. 12569810

[159] Li GH , Zou Y, Xia RY. A clinical evaluation of effectiveness of basic fibroblast growth factor in promoting wound healing. Herald of Medicine. 2002;21:215–7.

[160] Li HY . Effect of Genetime on wound healing after operation of anal diseases. China Prac Med. 2016;11:201–2.

[161] Fu Y , Jia ZX, Yan JR. RhEGF for clinical observation of postoperative wound healing of anal fissure. World Latest Medicine Information (Electronic Version). 2015;15:76–7.

[162] Qi HP , Zhang TQ, Zhao FL. Application of Genetime in postoperative wound infection. Modern Journal of Integrated Traditional Chinese and Western Medicine. 2009;18:3298–9.

[163] Li Y , Wei H. Observation of the use of recombinant human epidermal growth factor on cosmetic surgery wound. China Phamacist. 2012;15:694–5.

[164] Li TC , Qu D. Cosmetic plastic debridement and suture combined with recombinant human epidermal growth factor in the treatment of maxillofacial trauma. Chinese Journal of Aesthetic Medicine. 2016;25:30–2.

[165] Fan XH , Deng Q, Tan KL, Wu WJ, Luo ZB, Zhang SF, et al. Clinical study of Qufu Shengji treatment for promoting wound surface healing after low-set simple anal fistula surgery. Modern Journal of Integrated Traditional Chinese and Western Medicine. 2011;20:3914–6.

[166] Deng Q . Clinical Research on Accelerating the Postoperative Wound Healing of Anal Fistula by the Method of Eliminating Slough and Promoting Tissue Regeneration. Guangzhou University of Chinese Medicine, 2008.

[167] Liu HY , Jiang T, Huang WL, Xiao WM, Lei Y, Gao HW. Effect of artificial dermis combined with Jinfu Ning on skin healing and bacterial detection rate of finger abdomen. Journal of Hainan Medical University. 2019;25:1319–27.

[168] Li ZC , Ye W. Clinical observation of Sanqi Shengji ointment preventing skin flap necrosis. China Pharmacy. 2015;26:2806–8.

[169] Meng SY , Zhao P, Ma YN, Xu WH. Clinical effect of rh-aFGF on deep second degree burn wounds after early Escharectomy. Electronic Journal of Clinical Medical Literature. 2019;6:43.

[170] Huang WG , Zhan Y, Chen XH, Ren N, Zhou ZZ, Ying H, et al. Clinical study on the accelerative effect of externally used recombinant human basic fibroblast growth factor on wound healing after anal fistula surgery. Sichuan Medical Journal. 2018;39:925–31.

[171] He FJ . Observation of curative effect by recombinant human basic fibroblast growth factor for external use in the treatment of traumatic wound. Chin J Mod Drug Appl. 2015;9:30–1.

[172] Long Q , Li J, Wen Y. The clinical study of effect of recombinant bovine basic fibroblast growth factor gel combined with extract of Amebia Euchroma Tohnst yarn in accelerating effect wound healing anal fistula Postoperation. Jie Zhi Chang Gang Men Wai Ke. 2014;20:386–8.

[173] Guo Q , Sun YS. Application of recombinant bovine basic fibroblast growth factor in dressing change of wound after anal fistula operation. China Rural Health. 2018;21:77–9.

[174] Li ZB , Song LF, Guo XF, Shen W, Zhao ZQ. Effect of recombinant human epidermal growth factor gel on the recovery of the wound in older patients with mixed Hemorrhoids after operation. Jie Zhi Chang Gang Men Wai Ke. 2018;24:56–60.

[175] Jiang AL , Ma JY. Effect of recombinant human epidermal growth factor spray on large area skin abrasions caused by trauma. Dang Dai Hu Shi. 2018;25:73–4.

[176] Liu S , Qian HJ, Chen Z. Comparison of therapeutic effects of recombinant human epidermal growth factor and infrared radiation on children maxillofacial wound healing. China Medical Cosmetology. 2018;8:37–40.

[177] Liao M , Chen XY. Clinical application of recombinant human epidermal growth factor after anal fistula operation. Med Inform. 2008;21:2301–3.

[178] Lu H , Qin J, Wei KN. Prospective study of recombinant human epidermal growth factor on prevention of urinary fistula after hypospadias repair operation. Henan Journal of Surgery. 2017;23:10–2.

[179] Huang Y , Zhou JY, Li Y, Qi YH. Clinical observation of recombinant human epidermal growth factor in the treatment of mustard gas second degree skin injury. Med J Chin PLA. 2004;29:178.

[180] Lin L , Liu W, Wang CM, Li HC, Xu RZ. Treatment effect of recombinant human basic fibroblast growth factor-assisted cosmetic suture technique on emergency open traumatic wounds. Lin Chuang Yi Xue Gong Cheng. 2019;26:821–2.

[181] Liu JN , Wang W, Jia B. A randomized clinical study of recombinant human acidic fibroblast growth factor in the treatment of open wounds. Journal of Hebei Medical University. 2018;39:714–6.

[182] Jiang WM . Clinical observation on 35 cases of vitiligo treated with autologous epidermal transplantation combined with traditional Chinese and western medicine. Southern China Journal of Dermatovenereology. 2006;13:106–7.

[183] Sun CQ , Yang LH, Yu GZ, Yuan SK. Efficacy of bevacizumab combined with mupirocin in the treatment of Condyloma Acuminatum after CO2 laser surgery. China Prac Med. 2017;12:127–8.

[184] Sun RP , Zhao LK, Sun J, Ma JY, Li DJ, Li M, et al. Clinical observation of rh-aFGF application after early Escharectomy on deep second degree burn wound. Hebei Medical Journal. 2011;33:2144–5.

[185] Sun RP , Zhao LK, Sun J. CLinical observation of tangential excision of eschar and application rh-aFGF on limbs deep second degree burn wound. Chinese Journal of Aesthetic Medicine. 2014;23:5.

[186] Sun YL , Zhao WH, Zhang L. Rb-bFGF on healing of wound after operation for anal fistula. Chinese Journal of Practical Nervous Disease. 2009;12:74–5.

[187] Shi CF , Zhao ZL. Effect observation of recombinant human epidermal growth factor gel for wound repair after Freckle & Mole Laser Surgery. China Medical Herald. 2016;13:134–6.

[188] Shi ZY , Wang YQ. The effect on using of recombinant human epidermal growth factor hydrogel after anal fissure operation. Lin Chuang Tao Lun. 2012;50:126–7.

[189] Teng ZH , Wang YX, Xue WY, Zhu X, Li W, Qi JC, et al. Observation on the effect of recombinant human epidermal growth factor gel in urethral fistula after hypospadias operation. Hebei Medical Journal. 2015;37:1372–4.

[190] You S . Clinical Effect of Chlorophyll Derivatives on Postoperative Wound Healing of Low-order Simple Anal Fistula. North China University of Science and Technology, 2019.

[191] Wang SX . Application of rb-bFGF combined with wet dressing in hand mechanical injury. Journal of Yanan University. 2018;16:100–2.

[192] Wang LW , Wu CX, Wang YG. Effect of the application of recombinant human acidic fibroblast growth factor on wound healing and scar after procedure for prolapse and Hemorrhoids. China Medical Herald. 2014;11:56–9.

[193] Wang ZR , Liu T, Xu X, Li J, Lu K, Li ZY. Clinical effect of basic fibroblast growth factor on the healing of the skin wound. Chin Hosp Pharm J. 2008;28:638–40.

[194] Wen SJ , He LF, Huang T. Observation on the effect of Genetime solution in the treatment of infectious wound. Journal of Qilu Nursing. 2005;11:1548.

[195] Wanng GL . Clinical observation of two dressing changes in the treatment of diabetic skin Suppurative infection. Diabetes New World. 2016;69–70.

[196] Xu MC . Clinical study of cosmetic surgery debridement suture combined with recombinant human epidermal growth factor in the treatment of maxillofacial trauma. China Prac Med. 2019;14:2.

[197] Yao MY , Huang X, Guo QH. Prospective randomized controlled clinical trial of rhaFGF on cure of Cesarean incision on patients with highly infective risk factors. China Modern Doctor. 2014;52:26–31.

[198] Wu YJ , Nie M, Li DC, Jiang LH, Tian YY, Ying Y, et al. Effect of human Reconbinant fibroblast growth factor on wound healing in urology minimally invasive surgery. Infect Inflamm Rep. 2016;17:32–3.

[199] Wang JD , Yang WZ, Deng TT, Hua XK, Peng LF, Zhang ZY. Effect of recombinant bovine basic fibroblast growth factor on the healing of mixed Hemorrhoids. Journal of External Therapy of TCM. 2018;27:4–5.

[200] Wu ZX , Shou XM. Effect of recombinant bovine basic fibroblast growth factor on surgical wound. Modern Journal of Integrated Traditional Chinese and Western Medicine. 2004;13:2277–8.

[201] Xu H , Sun DS, Hu YY. Application of recombinant bovine basic fibroblast growth factor in wound healing. Chinese Journal of Trauma. 2000;16:344.

[202] Wei D . Effect of recombinant human epidermal growth factor combined with Beifuxin on the incision healing of facial plastic surgery. Journal of Chengdu Medical College. 2017;12:4.

[203] Xie YM , Chen MH, Ji Y, He YB. Clinical observation of recombinant human epidermal growth factor gel external use combined with Chinese medicine sit bath treatment for promoting wound surface healing after anal fistula surgery. Journal of New Chinese Medicine. 2013;45:58–60.

[204] Wu XZ , Xia JY, Chen SH. Study on the effect of recombination human epidermal growth factor on wound healing of anal fissure. Da Chang Gang Men Bing Wai Ke Za Zhi. 2004;10:100–2.

[205] Wang XH , Zhao XM, Zhao ZJ. The application of rhEGF in the Repairment of wound. Chinese Journal of Aesthetic Medicine. 2014;23:175–6.

[206] Wu XJ , Sun YF, Liu Y. A multiple Center, randomized, controlled trial of recombinant human acidic fibroblast growth factor in promoting wound healing after anal fissure surgery. Chin J Clinicians (Electronic Edition). 2013;7:11321–4.

[207] Zhi XY , Chen XS, Zeng XX. Observation on the effect of recombinant epidermal growth factor on grade II trauma. China Trop Med. 2007;7:70–1.

[208] Zhu SR , Wang XL, Tang GX, Tao XJ, Ji YX. Effect of rhEGF on wound healing after combined radical operation of oral cancer. Lin Chuang Kou Qiang Yi Xue Za Zhi. 2006;2:107–8.

[209] Zhang C , Chai SQ, Ma BB. Clinical observation of rhEGF in the treatment of severe bruising and contusion wounds. Journal of Dali University. 2015;14:47–9.

[210] Zhong XD , Lin HH, Chu ZH. Clinical observation of recombinant human epidermal growth factor in wound healing after Heamorrhoids surgery. Lingnan Modern Clinics in Surgery. 2015;15:166–8.

[211] Zhai YD , Cai N, Wei X. Treatment of 45 cases of anal fistula with recombinant bovine basic fibroblast growth factor. Journal of Qiannan Medical College for Nationalities. 2010;23:176–7.

[212] Zhang MF , Xu CP. Effect of basic fibroblast growth factor on promoting wound healing in acute sports injury. Fujian Sports Science and Technology. 2007;26:34–5.

[213] Zhang SH , Han CY. Effect of basic fibroblast growth factor on wound healing after CO2 laser surgery. Chin J Dermatol. 2001;34:1.

[214] Zhou J , Wu JX, Liu LX, Li JH, Wang YF, Kong LY. Effect of Longzhu ointment combined with Jinxuan Zhike fumigation powder on wound healing after perianal abscess operation. Herald of Medicine. 2011;30:1600–1.

[215] Mei JC , Chen Z, Deng FM, Xiao R, Chen B. Cosmetic plastic debridement and suture combined with recombinant human epidermal growth factor in the treatment of maxillofacial trauma. Modern Medicine and Health Research. 2019;3:67–70.

[216] Zhang RH , Tian ZB. Randomized controlled trial on application of recombinant basic fibroblast growth factor and compound four yellow liquid wound dressing for pollution wound treatment. China Modern Medicine. 2012;19:60–1.

[217] Zhu B , Dai L. Clinical study of recombinant human epidermal growth factor promoting wound healing after complex anal fistula operation. Chin J Mod Drug Appl. 2012;6:21–2.

[218] Zhou KL , Zou XJ. Effects of recombinant human epidermal growth factor on wound healing of perianal abscess after operation. J Clin Surg. 2015;23:286–7.

[219] Zhao K , Xu X. Clinical observation of recombinant human epidermal growth factor combined with thread drawing therapy in the treatment of high perianal abscess. Technique Communication. 2019;22:213–5.

[220] Zhu WZ . Effect and cost comparison of recombinant human basic fibroblast growth factor on wound healing. Zhong Guo Xiang Cun Yi Yao Za Zhi. 2007;14:28–9.

[221] Zhang HK . Effect of recombinant human acidic fibroblast growth factor on wound healing and scar of mixed Hemorrhoids after Hemorrhoidectomy. Chin Med J Metall Indus. 2019;36:604–5.

[222] Zhang ZM . Beifuji for promoting healing of wound after operation for Hemorrhoids. Chin J Coloproctol. 2004;24:8–9.

[223] Yun J , Yang GG, Liu ZY. Impact of basic fibroblast growth factor on wound surface healing of perianal infection. Chinese J Coloproctol. 2007;27:2.

[224] Huang W . Effects of recombinant human epidermal growth factor on wound healing of perianal abscess after operation. Chin J Prim Med Pharm. 2017;24:2026–9.

[225] Xu Q . Observation on the effect of epithelial growth factor in the superficial trauma wound care. Chinese Journal of Trauma and Disability Medicine. 2017;25:30–1.

[226] Zhang B , Han YM. Clinical analysis of efficacy of Shengji Baiyu Gao on wound-surface healing after perianal abscess operation. Chin J Coloproctol. 2017;37:2.

[227] Luo T . Clinical Effect of Recombinant Human Basic Fibroblast Growth Factor for External Use on Wound Healing after Lumbar Surgery. Southwest Medical University, 2018.

[228] Wang DX . Effect of external using recombinant human granulocyte-macrophage Colony Stimiulating factor gel on wound-surface healing after anal fistula surgery. Chin J Coloproctol. 2016;36:23–5.

[229] Sun LH , Song Y, Hua N. Clinical observation of recombinant human epidermal growth factor in the repair of auricle skin defect. Chin J Postgrad Med. 2010;33:66–7.

[230] Fu XB , Shen ZY, Chen YL, Xie JH, Guo ZR, Zhang ML, et al. Recombinant bovine basic fibroblast growth factor accelerates wound healing in patients with burns, donor sites and chronic dermal ulcers. Chin Med J (Engl). 2000;113:367–84. 11775238

[231] Ono I , Akasaka Y, Kikuchi R, Sakemoto A, Kamiya T, Yamashita T, et al. Basic fibroblast growth factor reduces scar formation in acute incisional wounds. Wound Repair Regen. 2007;15:617–23. 17971006 10.1111/j.1524-475X.2007.00293.x

[232] Yan DX , Liu S, Zhao XC, Bian HJ, Yao XW, Xing JP, et al. Recombinant human granulocyte macrophage colony stimulating factor in deep second-degree burn wound healing. Medicine. 2017;96. 10.1097/MD.0000000000006881 PMC5459702 28562537

[233] Lin Y , Chen MH, Ding FF, Wang RX, Liang ZQ, Meng CY, et al. Study of the use of recombinant human granulocyte-macrophage colony-stimulating factor hydrogel externally to treat residual wounds of extensive deep partial-thickness burn. Burns. 2015;41:1086–91. 25703666 10.1016/j.burns.2014.12.004

[234] Akita S , Akino K, Imaizumi T, Hirano A. Basic fibroblast growth factor accelerates and improves second-degree burn wound healing. Wound Repair Regen. 2008;16:635–41. 19128258 10.1111/j.1524-475X.2008.00414.x

[235] Nie KY , Li PC, Zeng XQ, Sun GF, Jin WH, Wei ZR, et al. Clinical observation of basic fibroblast growth factor combined with topical oxygen therapy in enhancing burn wound healing. Chinese J Reparative and Reconstructive Surgery. 2010;24:643–6. 20632489

[236] Hayashida K , Akita S. Quality of pediatric second-degree burn wound scars following the application of basic fibroblast growth factor: results of a randomized, controlled pilot study. Ostomy Wound Manage. 2012;58:32–6. 22879314

[237] Wang SL , Ma JL, Chai JK, Zhou L, Liao ZJ, Huang YS, et al. Acceleration of burn wound healing with topical application of recombinant human epidermal growth factor ointments. Chinese J Reparative and Reconstructive Surgery. 2002;16:173–6. 12569689

[238] Wang GY , Xia ZF, Zhu SH, Tang HT, Huan JN, Chen YL, et al. Clinical observation of the Long-term effects of rh-EGF on deep partial-thickness burn wounds. Chin J Burns. 2003;19:167–8. 12921622

[239] Wang ZY , Zhang Q, Liao ZJ, Han CM, Lv GZ, Luo CQ, et al. Effect of recombinant human granulocyte-macrophage Colony stimulating factor on wound healing in patients with deep partial thickness burn. Chin J Burns. 2008;24:107–10. 18785409

[240] Yan H , Chen J, Peng X. Recombinant human granulocyte-macrophage colony-stimulating factor hydrogel promotes healing of deep partial thickness burn wounds. Burns. 2012;38:877–81. 22652468 10.1016/j.burns.2012.02.001

[241] Zhang LP , Chen J, Han CM. A multicenter clinical trial of recombinant human GM-CSF hydrogel for the treatment of deep second-degree burns. Wound Repair Regen. 2009;17:685–9. 19769721 10.1111/j.1524-475X.2009.00526.x

[242] Zhang Y , Wang T, He JG, Dong JS. Growth factor therapy in patients with partial-thickness burns: a systematic review and meta-analysis. Int Wound J. 2016;13:354–66. 25040572 10.1111/iwj.12313 PMC7949794

[243] Abdelhakim M , Lin X, Ogawa R. The Japanese experience with basic fibroblast growth factor in cutaneous wound management and scar prevention: a systematic review of clinical and biological aspects. Dermatol Ther (Heidelb). 2020;10:569–87. 32506250 10.1007/s13555-020-00407-6 PMC7367968

[244] Briquez PS , Hubbell JA, Martino MM. Extracellular matrix-inspired growth factor delivery Systems for Skin Wound Healing. Adv Wound Care (New Rochelle). 2015;4:479–89. 26244104 10.1089/wound.2014.0603 PMC4505763

[245] Cohen MA , Eaglstein WH. Recombinant human platelet-derived growth factor gel speeds healing of acute full-thickness punch biopsy wounds. J Am Acad Dermatol. 2001;45:857–62. 11712030 10.1067/mjd.2001.117721

[246] Chipp E , Charles L, Thomas C, Whiting K, Moiemen N, Wilson Y. A prospective study of time to healing and hypertrophic scarring in paediatric burns: every day counts. Burns Trauma. 2017;5:3. 28116323 10.1186/s41038-016-0068-2 PMC5244545

