# Supplementary material for: The clinical effectiveness and safety of using epidermal growth factor, fibroblast growth factor and granulocyte-macrophage colony stimulating factor as therapeutics in acute skin wound healing: a systematic review and meta-analysis
Source: Burns Trauma. 2022 Mar 7;10:tkac002. doi: 10.1093/burnst/tkac002 (PMC8900703; doi:10.1093/burnst/tkac002)

Supplementary data

eFigure 1. Comparative meta-analysis of the healing rate of superficial second degree burn wounds.

eFigure 2. Comparative meta-analysis of the healing rate of deep second degree burn wounds.

eFigure 3. Comparative meta-analysis of the infection rate of superficial second degree burn wounds.

eFigure 4. Comparative meta-analysis of the infection rate of deep second degree burn wounds.

eFigure 5. Comparative meta-analysis of the adverse reaction of deep second degree burn wounds.

eFigure 6. Comparative meta-analysis of the healing rate of trauma and surgical wounds.

eFigure 7. Comparative meta-analysis of adverse reaction of trauma and surgical wounds.

eFigure 1. Comparative meta-analysis of the healing rate of superficial second degree burn wound.


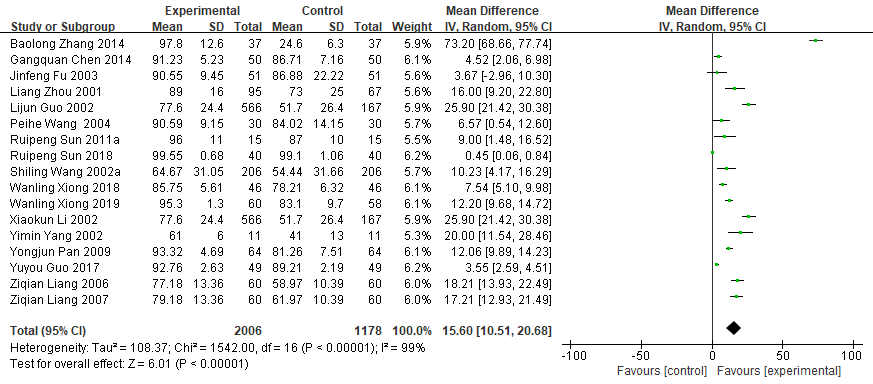


eFigure 2. Comparative meta-analysis of the healing rate of deep second degree burn wound.


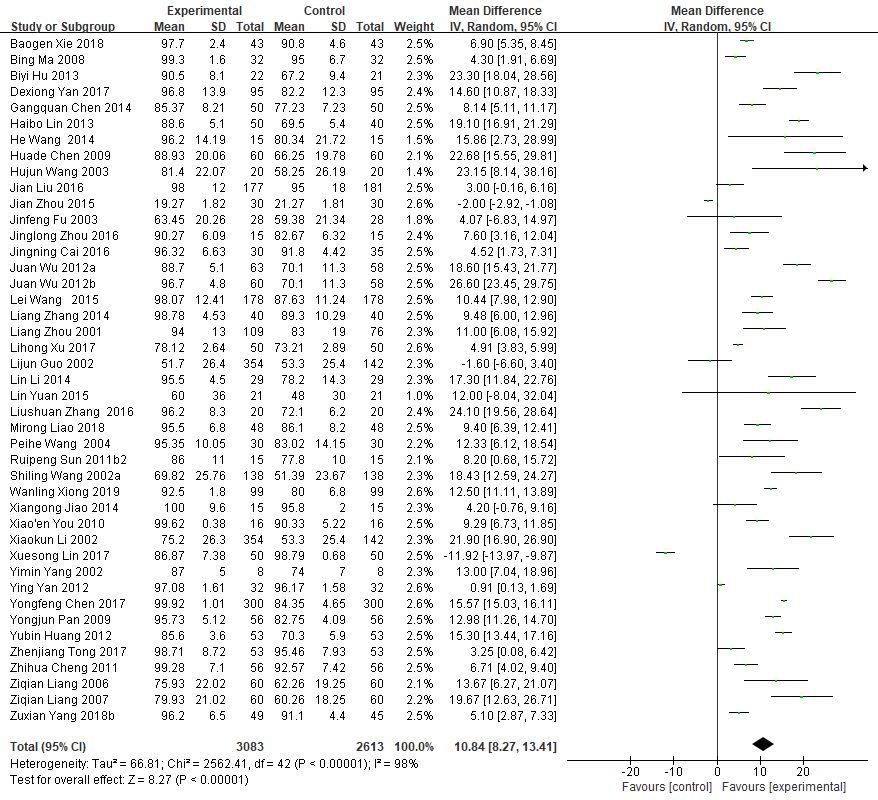


eFigure 3. Comparative meta-analysis of the infection rate of superficial second degree burn wounds.


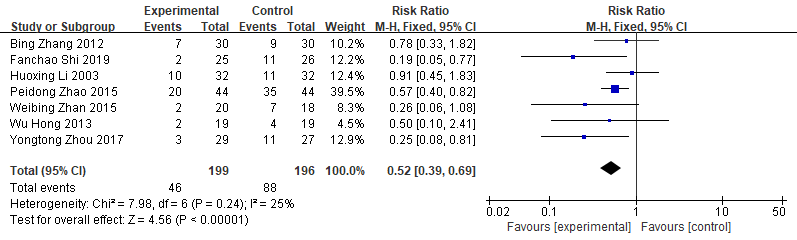


eFigure 4. Comparative meta-analysis of the infection rate of deep second degree burn wounds.


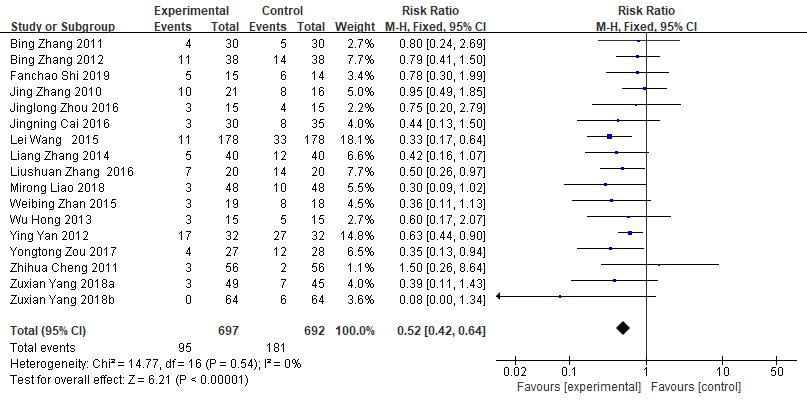


eFigure 5. Comparative meta-analysis of the adverse reaction of deep second degree burn wounds.


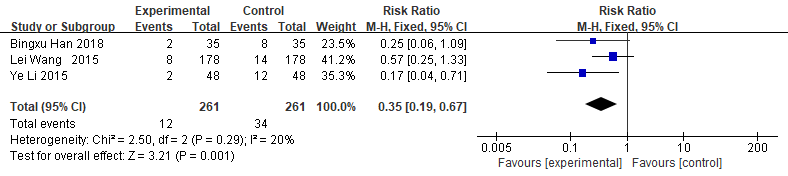


eFigure 6. Comparative meta-analysis of the healing rate of trauma and surgical wounds.


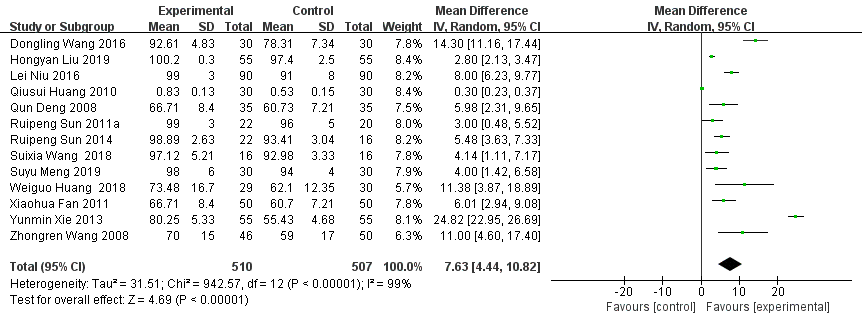


eFigure 7. Comparative meta-analysis of adverse reaction of trauma and surgical wounds.


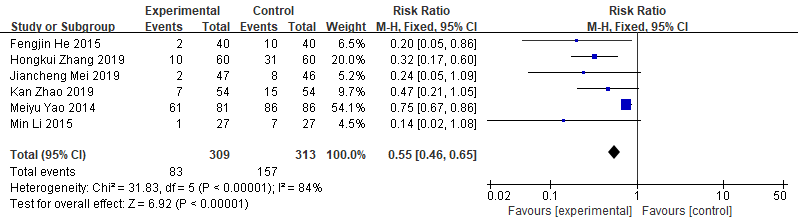

Supplement: Supplementary_data_tkac002 [file supplementary_data_tkac002.docx]
